# Anion-Assisted Glycosylation of Galactose: A Computational Study

**DOI:** 10.1021/acs.joc.5c02404

**Published:** 2025-12-03

**Authors:** Kerli Tali, Kaarel Erik Hunt, Kadri Kriis, Irina Osadchuk, Tõnis Kanger

**Affiliations:** Department of Chemistry and Biotechnology, 54561 Tallinn University of Technology, Akadeemia tee 15, 12618 Tallinn, Estonia

## Abstract

Glycosylation reactions and their chemistry are a field of significant contemporary importance. In this paper, a β-selective glycosylation reaction assisted by 2,6-di-*tert*-butylpyridinium tetrafluoroborate was experimentally performed, and its mechanism was computationally explained. Calculations showed that the first step of the reaction – substitution – goes through an S_N_2 mechanism and is the rate-determining step. Conformational analysis and transition state modeling showed that the substitution is catalyzed by the BF_4_^–^ anion, while the cation does not participate. *i*PrOH and trichloroacetamide (TCA) present in solution can significantly decrease the energy of the substitution step. This knowledge has then been applied to two saccharide glycosylation reactions described previously by other research groups, where the calculated reaction barriers were found to be too high for the experimental conditions given. Taking activators into account in the system in both cases allowed us to find transition states with significantly lower energy, which better correspond to stated experimental conditions.

## Graphical abstract

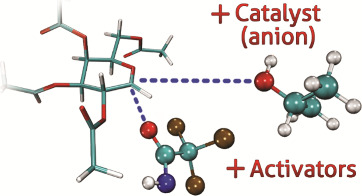

## Introduction

Carbohydrates are essential biomolecules, and their chemistry is an emerging field of significant contemporary importance. Their essential feature is stereochemistry at the anomeric carbon atom. For example, in the glycosylation reactions that are important for obtaining many biologically active glycoconjugates, a new stereocenter is formed.^1,2^ Stereoselective glycosidic bond formation, i.e., synthesis of α- or β-anomers, is vital for many different applications, such as vaccine and drug discovery.^1,3^ Due to the innate molecular complexity of carbohydrates, there is no universal method for their derivatization. Instead, there are a plethora of methods for chemo- and site-selective transformations of carbohydrates.^4^ In order to find more efficient synthetic methods, an understanding of the reaction mechanism is vital. Mechanistically, glycosylation reactions involving a triflate activating agent have received a widespread attention so far.^5–7^ However, other organocatalysts have also shown promising results in achieving high stereoselectivity; for example, pyridinium salt catalysts have been shown to give 1,2-*trans* glycosylation product with high selectivity in the case of 2,3,4,6-tetra-*O*-benzyl-d-glucopyranosyl trichloroacetimidate.^8^ A computational investigation of this system, in which the cation and anion were considered separately, found barriers of 36.8 and 44.9 kcal/mol, respectively.^8^ Ghorai *et al.* ^9^ found barriers of 33.0 and 55.2 kcal/mol for 2,3,4,6-tetra-*O*-benzyl-d-glucopyranosyl trichloroacetimidate glycosylation reaction with methanol catalyzed by protonated phenanthrolinium BF_4_^–^ salt. High activation energies of these reactions led us to investigate further the mechanism of strained ion pair catalysis. For this purpose, the glycosylation of a 2,3,4,6-tetra-*O*-acetyl-d-galactopyranosyl trichloroacetimidate donor with *i*PrOH as an acceptor in the presence of 2,6-di-*tert*-butylpyridinium tetrafluoroborate (catalyst **1**) was chosen as a model reaction (Scheme 1).

**Scheme 1:**
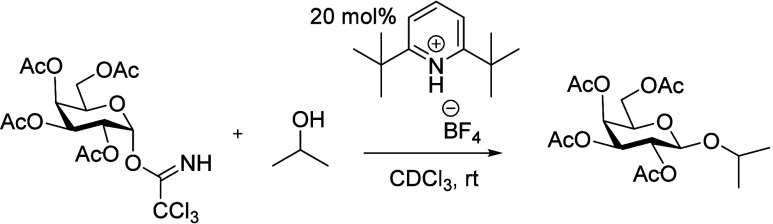
Reaction System Investigated

## Results and Discussion

Experimentally, the reaction was complete within 1 h at rt under an Ar atmosphere in the presence of 20 mol % of catalyst **1** in anhydrous CDCl_3_. The reaction with this new catalyst selectively afforded the 1,2-*trans-*product (β-anomer) in a nearly quantitative yield. When catalyst **1** is not present in the reaction mixture, the product is not formed even after reaction for 7 days of reaction time.

### Conformational Search

To understand catalyst-saccharide interactions and get initial geometries for further reaction mechanism modeling, a conformational search was performed using the CREST program and the GFN-FF force field that has previously demonstrated good abilities in the description of conformational landscape,^10–13^ followed by Gaussian 16 calculations using SMD_(chloroform)_-B3LYP-D3­(BJ)/6-31++G**//B3LYP-D3­(BJ)/6-31+G* level of theory (the DFT method successfully used in conformation description).^14–17^

To speed up calculations, the reacting system was split into three starting models:

1. Saccharide + catalyst (pyridinium cation + tetrafluoroborate anion);
2. Saccharide + pyridinium cation + *i*PrOH;
3. Saccharide + tetrafluoroborate anion + *i*PrOH.

For the first model system (saccharide + catalyst (pyridinium cation + tetrafluoroborate anion)), conformation analysis revealed the presence of two main conformers, **1** and **2** (Figure 1 and Table S1). In the lowest-energy **conformer 1** with a Boltzmann population of 64.8% at 298 K, the catalyst is placed on the opposite side from the anomeric carbon. Topology analyses show that BF_4_^–^ anion has no H-bond with the cationic part of the catalyst. Also, there is no interaction between the catalyst and the anomeric carbon. In contrast, in **conformer 2**, with a population of 34.4%, the anionic and cationic parts of the catalyst are separated. The cationic part is situated on the opposite side from the galactose reaction center, and the BF_4_^–^ anion is situated close to the pyridine ring. This agrees with a study by Addanki *et al.*,^8^ where in the crystal structure of 2,4,6-tri-*tert*-butylpyridinium tetrafluoroborate, the anion was shown to be situated close to *tert*-butyl groups instead of forming an H-bond with the N–H group. Conformational search for the first model system proves the mobility of the BF_4_^–^ anion.

**Figure 1:**
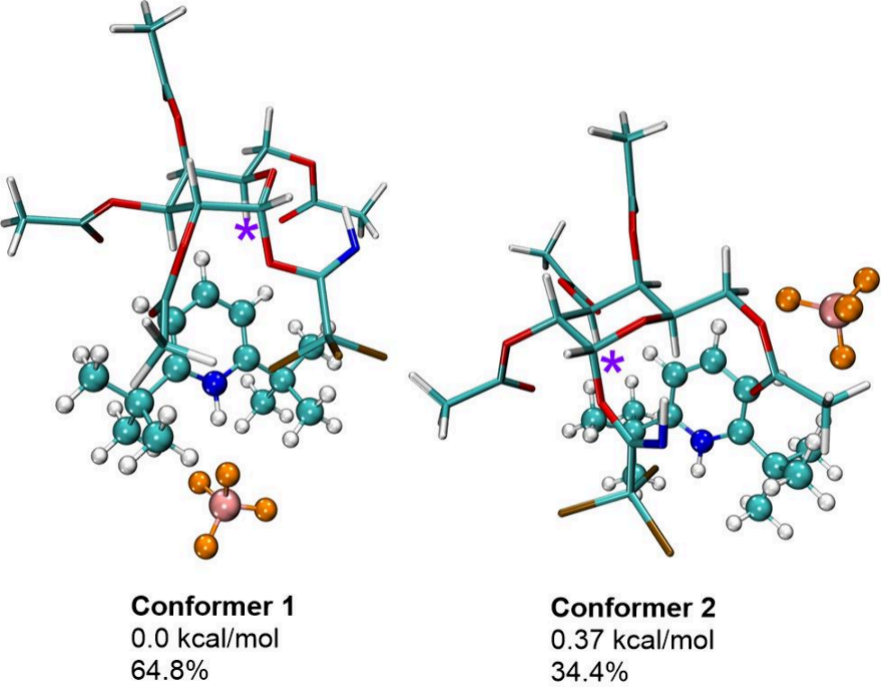
Lowest energy conformers for the saccharide + catalyst (pyridinium cation + tetrafluoroborate anion) model system. Anomeric carbon is marked with a purple asterisk.

It is also worth noting that the difference in energy of 0.4 kcal/mol lies within the limits of the accuracy of the DFT approach, and the content of **conformer 2** may be higher.^18–20^ In addition, we do not consider the possibility that conformers may be separated by low energy barriers, exclude dynamic effects, and use the simple Boltzmann averaging procedure to estimate the conformational population of noncovalently bound complexes. All these assumptions introduce additional uncertainty into the calculations.^21^

Conformational search for the second model system (saccharide + pyridinium cation + *i*PrOH) revealed the presence of two main **conformers 3** and **4** (Figure 2 and Table S2). The structures found are similar. In the lowest-energy **conformer 3** with a population of 57.7%, *i*PrOH has an H-bond with the third position acetoxy group, while in **conformer 4** with a population of 35.3%, with the second position acetoxy group. Herein, in both **conformers 3** and **4**, *i*PrOH forms an H-bond with the pyridinium part of the catalyst (Figure S1 and Table S3). It should be noted that in both found conformers of the saccharide + cation + *i*PrOH system, *i*PrOH is far away from the reaction center.

**Figure 2:**
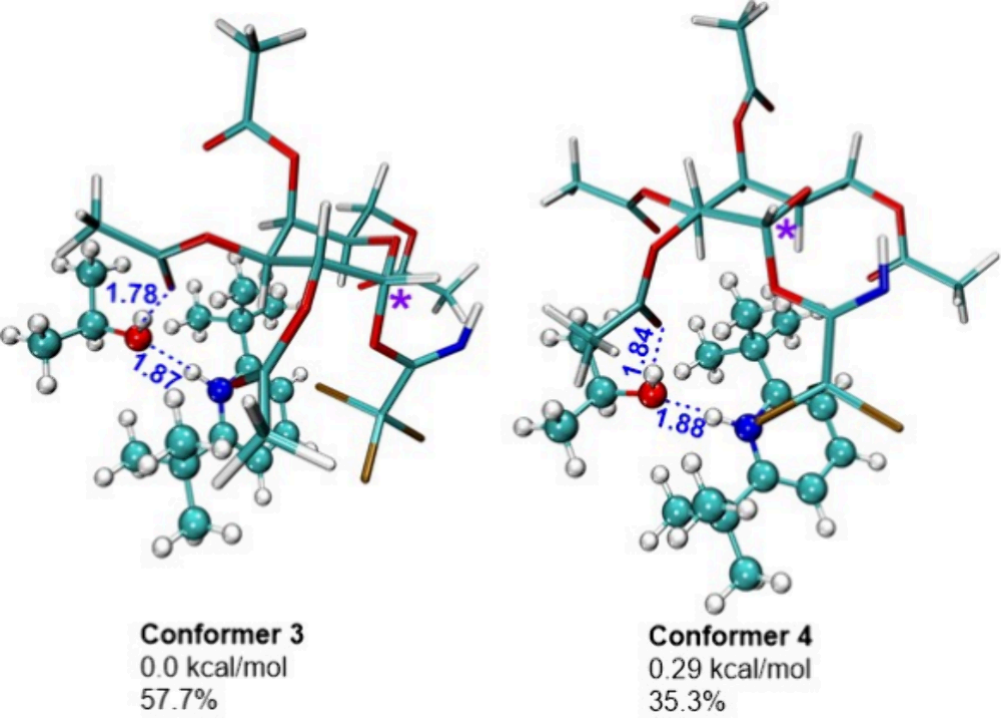
Lowest energy conformers for the saccharide + pyridinium cation + *i*PrOH model system. H-bonds are marked in blue, and distances are shown in Å. Anomeric carbon is marked with a purple asterisk.

Conformational search for the third model system (saccharide + anion + *i*PrOH) revealed the presence of six conformers with probability higher than 5% which make up about 90% of the conformational space (Figure 3 and Table S4). The differences in the relative energies of the six lowest energy conformers do not exceed 0.80 kcal/mol, and population varies from 25.9 to 6.7%. In the five lowest energy conformers, *i*PrOH is close to the reaction center with C–O a distance of 3.32–4.41 Å (Figure 3), and the biggest difference between **conformers 5–9** is the exact position of *i*PrOH and BF_4_^–^ anion. In **conformers 5**, **7**, and **9**, the BF_4_^–^ anion is placed between *i*PrOH and the leaving group, but in **conformers 6** and **8**, *i*PrOH is placed between the BF_4_^–^ anion and the leaving group. In all six conformers, the H–F distance is small (1.76–1.85 Å), so the H-bond between BF_4_^–^ and the acceptor is formed with H-bond strengths of 10.1–11.2 kcal/mol (Figure S1 and Table S3). In **conformer 5**, a H-bond is also formed between BF_4_^–^ and the leaving group with a strength of 7.3 kcal/mol, respectively. In contrast, **conformers 6, 7, and 9** do not form an H-bond between the leaving group and the BF_4_^–^ anion due to the increased distance. It should be noted that **conformer 8** is the only structure that has an H-bond between *i*PrOH and the leaving group. In contrast to conformers **5–9**, in conformer **10**, the distance between *i*PrOH and the reaction center is 4.91 Å, and the positioning of *i*PrOH makes the glycosylation reaction unlikely.

**Figure 3:**
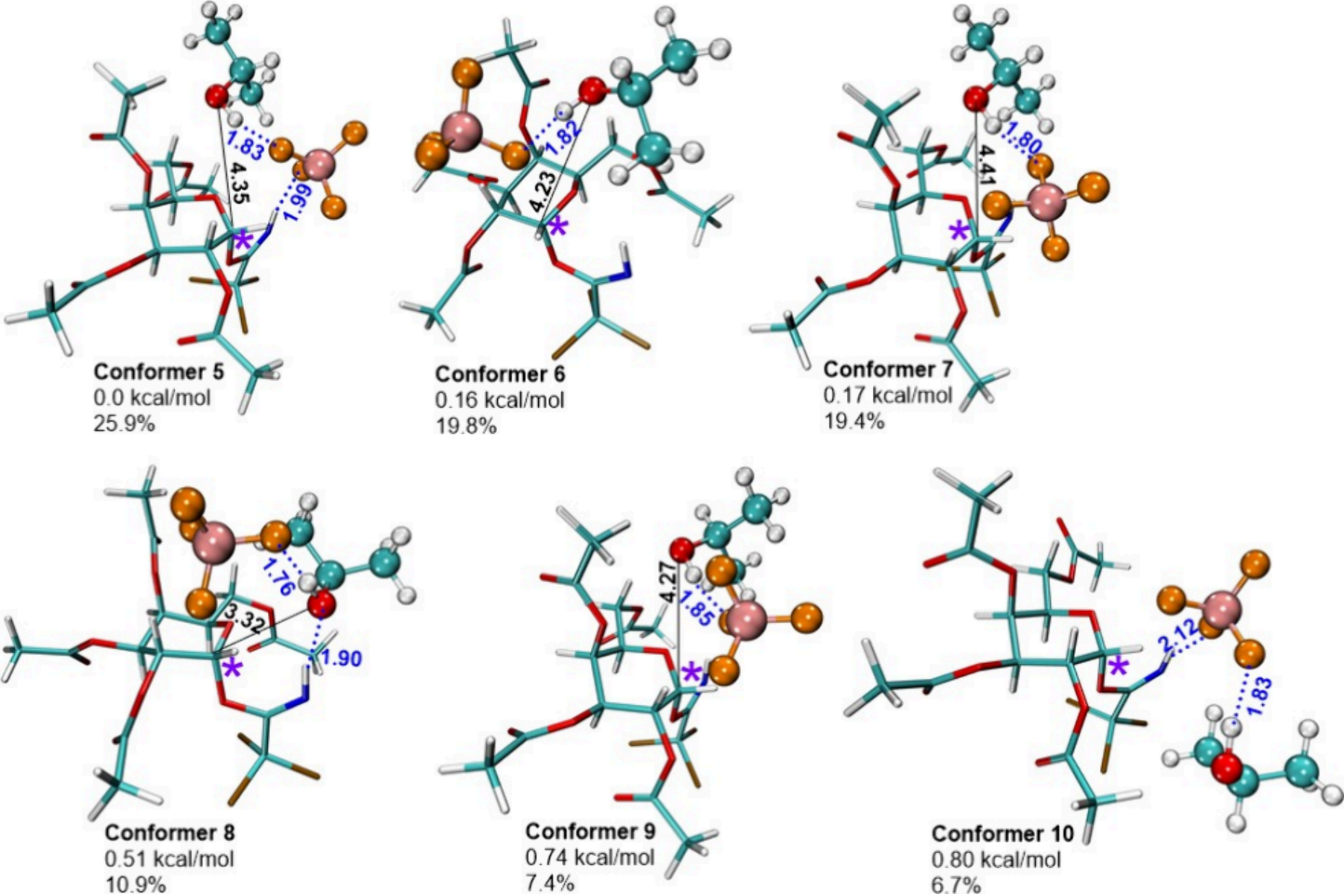
Six lowest energy conformers of the third model system (saccharide + tetrafluoroborate anion + *i*PrOH). H-bonds are marked in blue, distances are shown in Å. Anomeric carbon is marked with a purple asterisk.

To summarize, based on the conformational search performed for three models (1) saccharide + catalyst (cation + anion), (2) saccharide + cation + *i*PrOH, and (3) saccharide + anion + *i*PrOH, we conclude that the BF_4_^–^ anion is mobile and, with high probability, is not connected to the cationic part of the catalyst. Moreover, considering the geometries of conformers found, it is the BF_4_^–^ anion that catalyzes the reaction. At the same time, hydrogen bonds play an important role in determining the position of *i*PrOH and the BF_4_^–^ anion and stabilization of the system. This agrees well with previous computational and experimental studies.^22–25^ For this reason, **conformers 5** and **6** with populations of 25.9 and 19.8%, respectively, were further used as starting points for reaction modeling. **Conformer 7** was not used in reaction modeling despite its high probability because due to its similarity to conformer **6**, it is unlikely to lead to a different transition state.

### Reaction Mechanism

Mechanistically, the reaction under investigation is thought to broadly proceed through two main steps (Scheme 2): substitution and proton transfer.

**Scheme 2:**
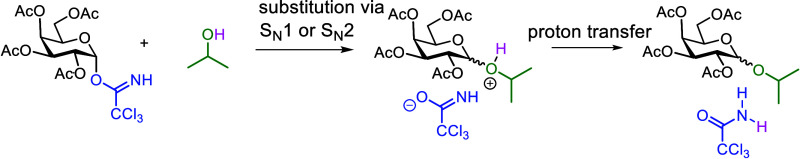
Two Main Steps of the Reaction

During substitution, a glycosidic bond is formed between the donor and the acceptor through either the S_N_1 or the S_N_2 mechanism (Scheme 3). Adero *et al.*,^26^ in their review article, have shown that glycosylation reactions take place on a continuum between S_N_1 and S_N_2 mechanisms. However, when glycosylation reaction mechanisms are studied, they are usually associated with S_N_1 or S_N_2 reaction types. In the S_N_1 reaction, an oxocarbenium ion is formed first, followed by the nucleophilic attack of the acceptor, leading to an anomeric mixture. In the S_N_2 reaction, substitution happens in a single step, and it leads to the inversion of configuration. The substitution is followed by proton transfer, where a proton from an acceptor is transferred to the leaving group.

**Scheme 3:**
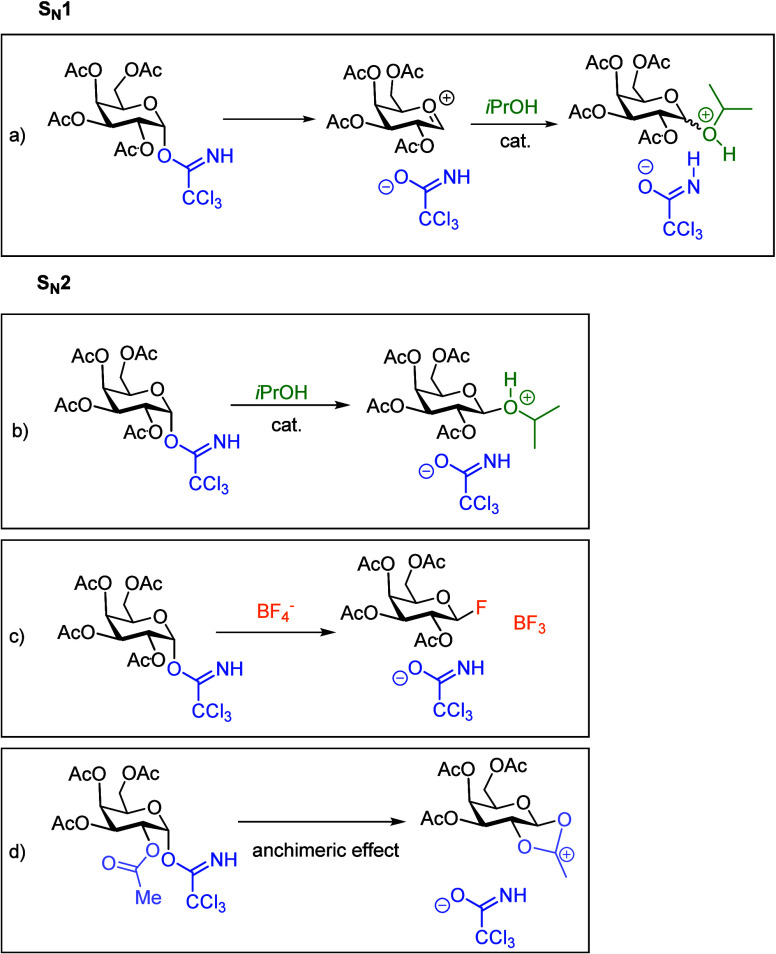
Possible Reaction Pathways^a^

a (a) S_N_1 mechanism; (b) β-anomer formation through S_N_2 mechanism; (c) β-Gal-F formation; (d) anchimeric effect of second position acetoxy group.

To study the reaction mechanism, the SMD_(chloroform)_-mPW1PW91-PFD/def2-TZVPP//mPW1PW91-PFD/def2-SVP method was selected as an optimal method for our modeled system. This choice was made based on benchmark studies of organic systems^27–41^ and on our performance test calculations. More information about the choice of method can be found in the Computational Details section.

#### Step 1: Substitution

##### S_N_1 Mechanism

S_N_1 substitution in glycosylation reactions and the formation of the oxocarbenium ion in glycosylation reactions have been investigated both experimentally and theoretically.^22–25^ It was shown that the stability of the oxocarbenium ion depends on the position of the substituents, pyranose ring conformation, and solvent. Thus, the oxocarbenium ion is much more likely to be formed in a polar solvent. In contrast, our reaction occurs in a relatively nonpolar solvent (chloroform), which is favorable for an S_N_2 substitution mechanism. This was confirmed by our calculations. A potential energy surface (PES) scan (at the mPW1PW91-PFD/def2-SVP level of theory) showed that the formed intermediate (INT) is 37.5 kcal/mol higher in energy compared to the reagents (Figures S2–S5). The optimization of the contact ion pair (S_N_1 intermediate) leads to the reagent geometry. Based on the results listed above, we decided that substitution in the considered conditions occurs through an S_N_2 mechanism.

##### S_N_2 Mechanism

If the reaction occurs through a S_N_2 mechanism, several pathways are possible. The first option is the reaction occurring in one step with *i*PrOH acting as a nucleophile (Scheme 3b). This is also supported by the structures obtained from a conformational search (**conformers 5**-**9**, Figure 3).

The second possibility is the formation of a glycosidic bond by a F^–^ anion derived from the BF_4_^–^ catalyst (Scheme 3c). In the case of triflate-promoted glycosylation reactions, the presence of a triflate-containing intermediate for an S_N_2 mechanism has been shown both experimentally and theoretically.^5,42,43^ However, our calculations showed that F^–^ glycosylation is unfavorable; the intermediate is 42.3 kcal/mol higher in energy than reagents (Figure S6).

The third possibility is neighboring group participation (Scheme 3d).^44,45^ While this is mostly associated with an S_N_1 reaction, there have been proposed glycosylation mechanisms that also use this in an S_N_2 reaction.^46^ The second position, OAc-group neighboring group participation, was found to have a barrier of 37.3 kcal/mol.

Based on all of the above, we concluded that the reaction occurs in one step with *i*PrOH acting as a nucleophile (Scheme 3b). This pathway was also supported by conformational search. As was said above, two different positions of the BF_4_^–^ anion are possible: most probable (56.7% – **conformers 5, 7,** and **9**), where the BF_4_^–^ anion is placed between *i*PrOH and the leaving group (Figure 3), and less probable (32.8% – **conformers 6** and **8**), where *i*PrOH is placed between the BF_4_^–^ anion and leaving group. The corresponding transition state of this step is marked **TS1,** which leads to **INT1**, where the glycosylation reaction has taken place, but intermediate 1 is still protonated.

Geometry of **TS1–1** (Figure 4) corresponds to a ground state where the BF_4_^–^ anion is placed between *i*PrOH and the leaving group. In **TS1–1**, similar to **conformer 5**, an H-bonded cycle between the pyranose ring, the leaving group, and the tetrafluoroborate anion is present. However, the pyranose ring adopts a half-chair conformation (^4^H_3_), in contrast to the reagent structure, where the pyranose ring has a chair conformation (^4^C_1_). The distance between the leaving group’s oxygen (O1) and the ring’s C1 is 2.51 Å, and between the acceptor’s oxygen (O2) and the ring’s C1–1.83 Å. The H-bond lengths between the anion and the acceptor and between the anion and the leaving group are given in Figure 4. **TS1–1** is 34.1 kcal/mol higher in energy than the reagents (Table S5). This is in good agreement with the barrier height of 32.8 kcal/mol proposed by Addanki *et al*.^8^ for a similar substitution catalyzed by the BF_4_^–^ anion. Moreover, it also aligns with the conclusion based on conformational search, which indicates that the cationic part of the catalyst does not participate in the substitution.

**Figure 4:**
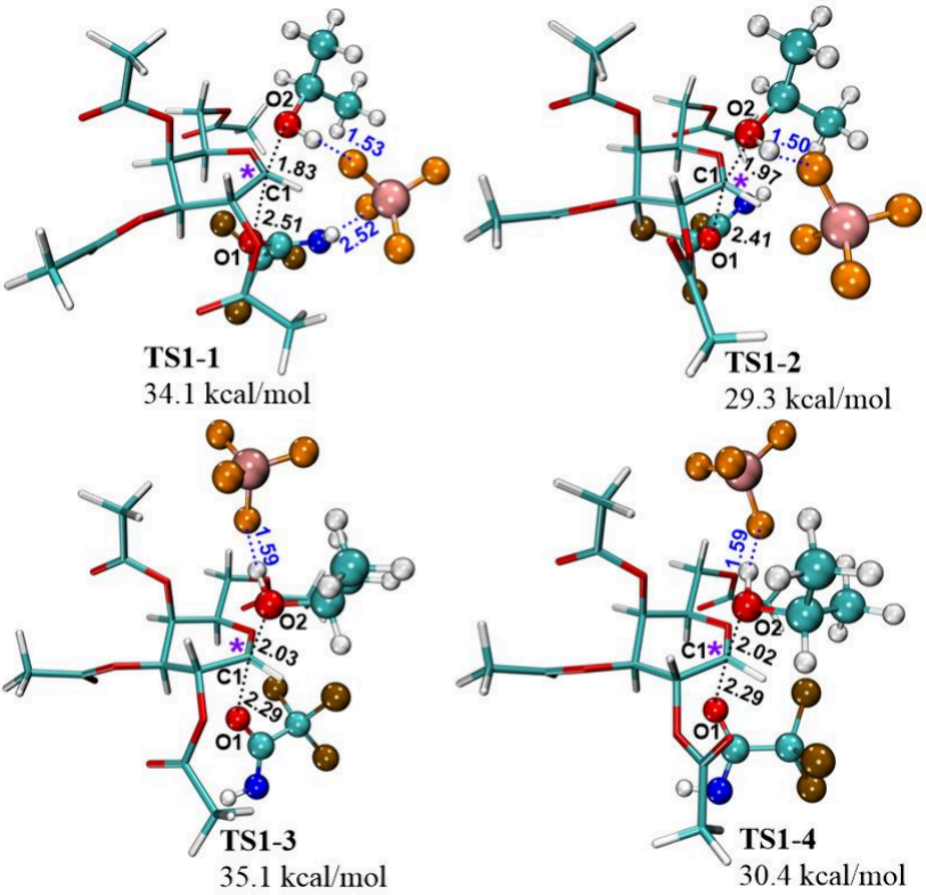
Possible TSs for S_N_2 substitution in one step. Formed H-bond lengths are given in Å, and the anomeric carbon is marked with a purple asterisk.

Interestingly, when the second position acetoxy group is rotated 180°, the energy of the TS decreases from 34.1 to 29.3 kcal/mol (Figure 4 **TS1–2** and Table S5). Since the calculated second position acetoxy group rotation barrier is 3.0 kcal/mol, the rotation is orders of magnitude faster than the glycosylation reaction and can easily take place (Figure S7).

**TS1–3** (Figure 4) corresponds to a less probable ground state where *i*PrOH is placed between the BF_4_^–^ anion and the leaving group, and the pyranose ring has the chair conformation (^4^C_1_) and is close in geometry to **conformer 8**. In **TS1–3**, like in **TS1–1** and **TS1–2**, the pyranose ring adopts the half-chair conformation (^4^H_3_), but in contrast to **TS1–1** and **TS1–2**, the BF_4_^–^ anion has an H-bond with the acceptor. **TS1–3** is 35.1 kcal/mol higher in energy than the reagents (Figure 4 and Table S5). Just as was the case for **TS1–1**, the rotation of the second position acetoxy group by 180° lowers the energy to 30.4 kcal/mol, resulting in **TS1–4** (Figure 4 and Table S5).

None of the located TSs have low enough energy for the glycosylation reaction to proceed at room temperature. It has been previously shown by several sources, both experimentally and computationally^47–49^ that hydrogen bonds with the catalyst are important in glycosylation reactions. It is possible that other H-bond donors present in the reaction mixture, such as *i*PrOH, and in the later part of the reaction, the forming side product, trichloroacetamide (TCA), could also decrease the energy of the transition state. Ghorai *et al.* ^9^ have experimentally proved that TCA plays a role in the glycosylation reaction mechanism in the case of trichloroacetimidate-containing donor.

Out of the located TSs for the first step, we chose two with the lowest energy (**TS1–2** and **TS1–4**) and tried to decrease their energy by using the influence of H-bond donor(s) to the leaving trichloroacetimidate group (Figure 5 and Table S6). To make the newly created TSs comparable in Gibbs free energies to the previously presented structures, we additionally calculated the corresponding reagent structures for each TS and used their energies as the zero point. The addition of one *i*PrOH molecule (marked by * per each *i*PrOH) reduced the energy of **TS1–2** from 29.3 to 27.5 (TS1–2*­(A)) or to 26.0 kcal/mol (TS1–2*­(B)), depending on the position of the *i*PrOH molecule (defined by A or B letters shown in Figure 5). Adding two *i*PrOH molecules to both A and B positions decreased the energy up to 22.4 kcal/mol (TS1–2**). The H-bond lengths are given in Figure 5C and the strengths in Table S8.

**Figure 5:**
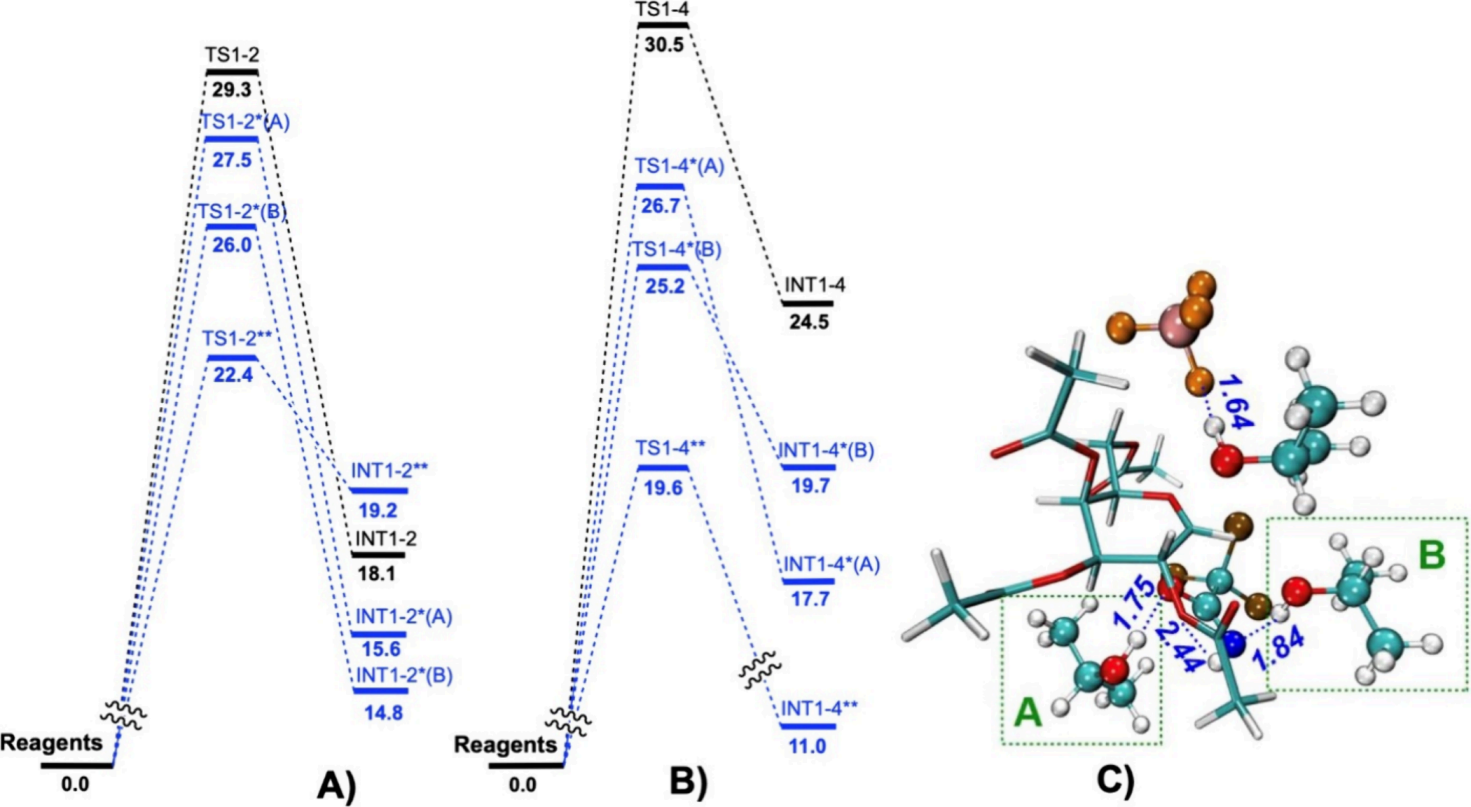
(A) Gibbs free energy in kcal/mol for **TS1–2** with (in blue) and without (in black) addition of *i*PrOH molecule(s). (B) Gibbs free energy of **TS1–4** with (in blue) and without (in black) addition of *i*PrOH molecule(s). (C) Geometry of TS1–4** structure where *­(A) and *­(B) mark the presence of *i*PrOHs in a certain position. Formed H-bonds are marked in blue and distances are shown in Å.

We also tried to decrease the **TS1–4** energy similarly. Addition of *i*PrOH molecule(s) reduced the energy from 30.5 to 26.7 kcal/mol in TS1–4*­(A), to 25.2 kcal/mol in TS1–4*­(B), and to 19.6 kcal/mol in TS1–4** (Figure 5B, Table S6). Herein, when catalyst BF_4_^–^ is removed from TS1–4**, the energy rises from 19.6 to 25.2 kcal/mol, showing that the presence of *i*PrOH alone is not enough for the reaction to proceed. This corresponds to our experimental observation that without the catalyst present, the glycosylation reaction does not proceed.

As was mentioned previously, TCA, as a H-bond donor, can participate in the reaction, especially in the later stages of the reaction. Addition of one TCA molecule (marked by ’ per TCA molecule) decreases the energy of **TS1–2’** from 29.3 to either 24.2 or 25.3 kcal/mol and from 30.5 to either 23.0 or 23.3 for **TS1–4’** (Figure 6, Table S7). Adding two TCA molecules decreases the barrier to 19.5 in the case of **TS1–2’’** and 17.7 kcal/mol in the case of **TS1–4’’** (Figure 6, Table S7). Having one *i*PrOH molecule and one TCA molecule as mediator would mean the barrier height of 24.6 for TS1–2*’ and 20.3 or 23.3 kcal/mol for TS1–4*’. Even though O–H hydrogen bonds are generally stronger than NH-H hydrogen bonds,^50^ in the studied system, the opposite situation is observed – HN-H hydrogen bonds being shorter than O–H bonds (Figure S8, Tables S8, and S9), providing lower-energy TSs. We associate this with the large differences in the partial charges. Thus, in *i*PrOH, Mulliken partial charges at oxygen atoms are −0.385 and −0.370 and at hydrogen atoms +0.250 and +0.207, but in TCA, −0.141 and −0.128 at nitrogen and +0.198 and +0.193 at hydrogen atoms.

**Figure 6:**
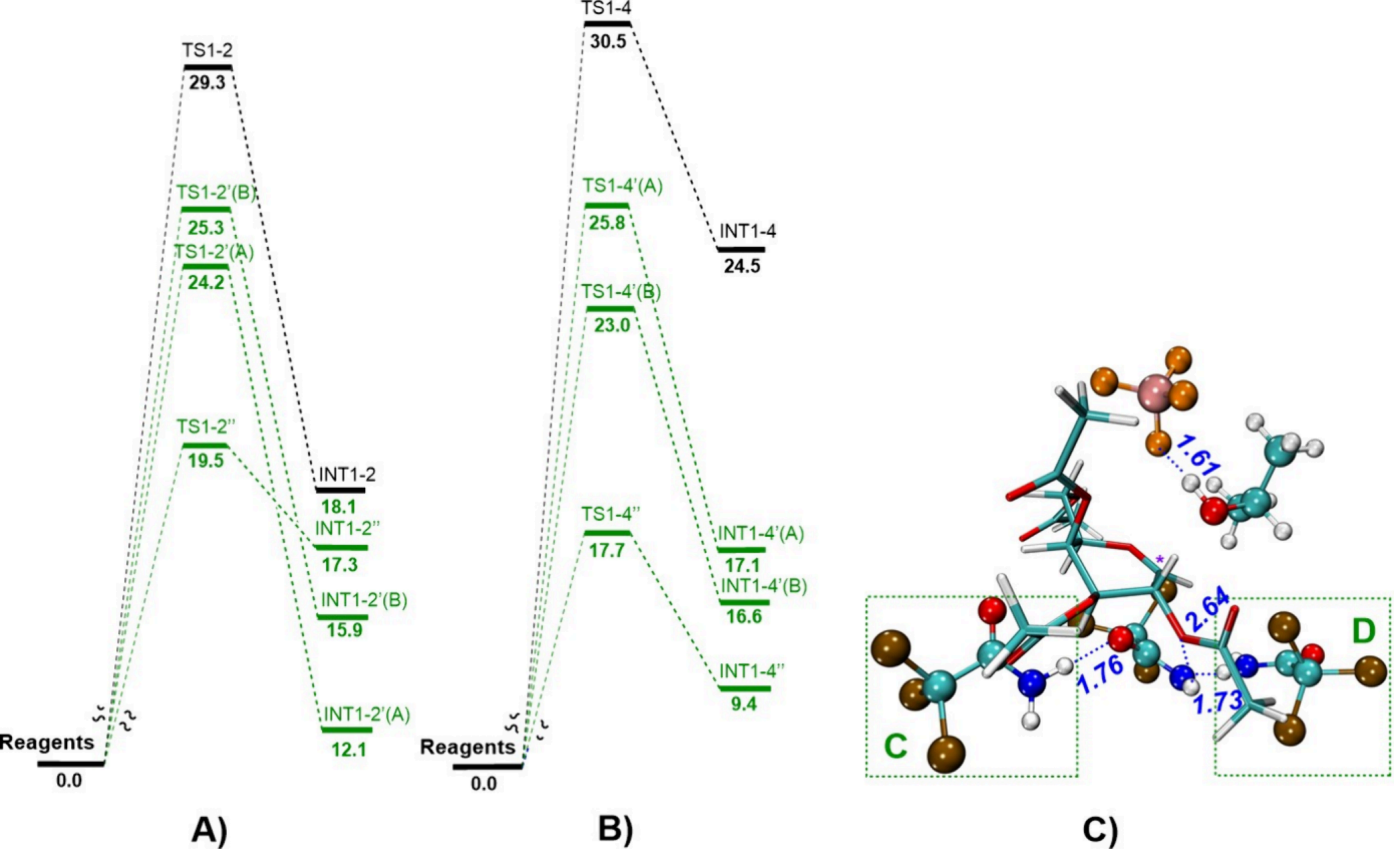
(A) Gibbs free energy in kcal/mol for **TS1–2** with (in green) and without (in black) addition of TCA molecule(s). (B) Gibbs free energy of **TS1–4** with (in green) and without (in black) addition of TCA molecule(s). (C) Geometry of **TS1–4”** where (C) and (D) mark the presence of TCA in a certain position. Formed H-bonds are marked in blue and distances are shown in Å.

#### Step 2: Proton Transfer

The second main step in the glycosylation reaction is proton transfer from the acceptor to the leaving group. There are two possibilities for how this can work: either with participation by the BF_4_^–^ catalyst or without. It is evident from the intermediate structures (INT1–4** and **INT1–4’’**) that proton transfer will occur through a mediator (either *i*PrOH or TCA). Therefore, there are four possibilities:

1) *i*PrOH mediator with BF_4_^–^ catalyst;
2) *i*PrOH mediator alone;
3) TCA mediator with BF_4_^–^ catalyst;
4) TCA mediator alone.

In model **system 1** (*i*PrOH mediator with the BF_4_^–^ catalyst), proton transfer occurs through the BF_4_^–^ catalyst. First, reorganization of **INT1** into **INT2** happens (Figure 7 (blue pathway), Tables S10 and S11, Figure S9). Then, the isopropyl group rotated through the C1–O2 bond with a barrier of 8.2 kcal/mol (**TS2**, 22.5 kcal/mol (vs reagent)), resulting in the formation of **INT3**. Further, a barrierless proton transfer occurs (Figure S10) from the protonated isopropyl group to the *i*PrOH mediator and simultaneously, *i*PrOH’s proton is transferred to the leaving group, forming the product. The reaction pathway is similar when TCAs are mediators instead of *i*PrOHs (**system 3**). However, in the case of TCA mediator with BF_4_^–^ catalyst, the proton transfer occurs with a barrier of 5.2 kcal/mol (15.0 kcal/mol (vs reagent)) (green pathway in Figure 7). Structures of intermediates and PES scans can be found from Figures S11 and S12, and H-bonds in Table S12.

**Figure 7:**
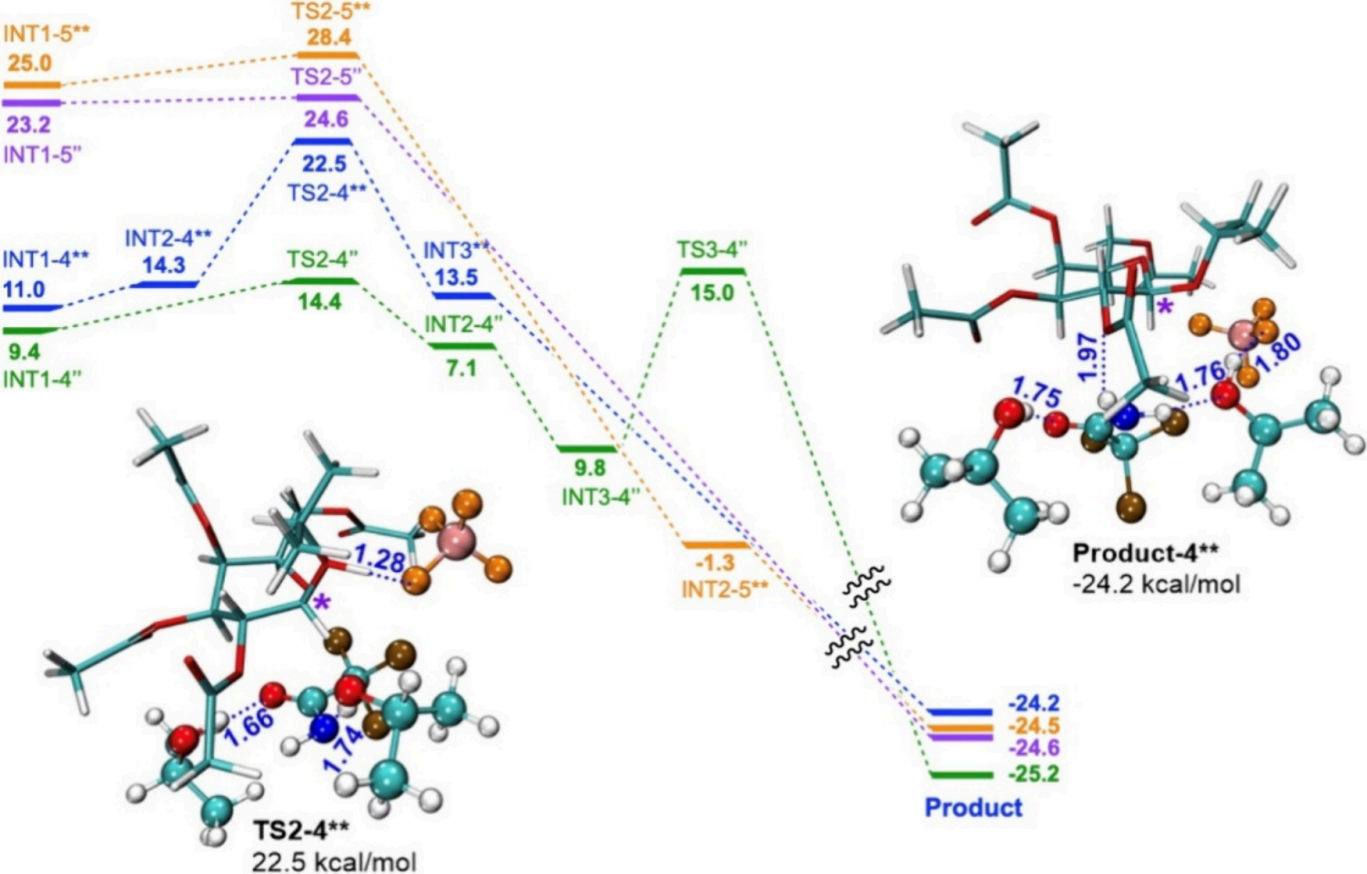
Energy profile for proton transfer. System 1 energies are given in blue, system 2 in orange, system 3 in green and system 4 in purple. Energies are given in kcal/mol.

When BF_4_^–^ is absent (**systems 2 and 4**), proton transfer occurs without a barrier (Figures S13 and S14). However, preceding isopropyl group rotation has a barrier of 3.4 kcal/mol (28.4 kcal/mol (vs reagent)) for **system 2** (Figure 7 – orange pathway, PES scan in Figure S15) and 1.4 kcal/mol (24.6 kcal/mol (vs reagent)) for **system 4** (Figure 7; purple pathway, PES scan in Figure S17, structures in Figure S18). Product energies differ slightly due to the positioning of activator molecules.

Based on these results, it can be said that in the proton transfer step the BF_4_^–^ anion facilitates preceding isopropyl group rotation and slightly inhibits the proton transfer.

### Mechanism of the Whole Reaction

The glycosylation reaction of 2,3,4,6-tetra-*O*-acetyl-d-galactopyranosyl trichloroacetimidate with *i*PrOH catalyzed by 2,6-di-*tert*-butylpyrimidinium BF_4_^–^ was found to proceed through an S_N_2 mechanism in the substitution/glycosylation step. This agrees with our experimental observation that a β-anomer is formed and the reaction is selective. The substitution step **(TS1)** is rate-determining and significantly affected by the presence of H-bond donors, since **TS1** is activated by either *i*PrOH or side product TCA molecules, depending on the stage of the reaction. Presence of *i*PrOH/TCA lowers the barrier from 30.5 to 19.6 kcal/mol (TS1–4**) and 17.7 kcal/mol (**TS1–4’’**), respectively (Figure 8).

**Figure 8:**
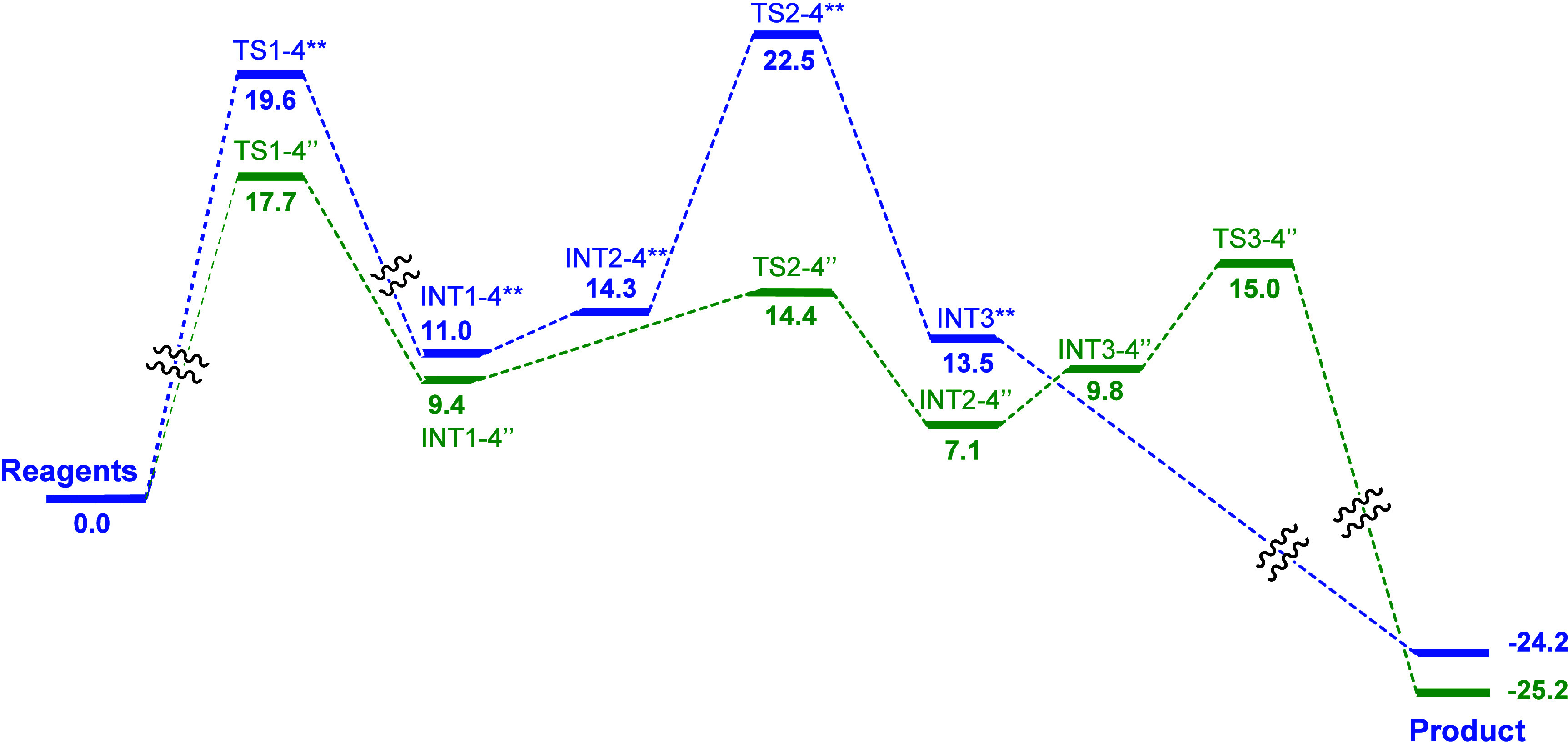
Energy profile for the whole reaction. Energies for the *i*PrOH stabilized pathway are given in blue, energies for TCA stabilized pathway in green. Energies are given in kcal/mol.

In the second main step of the glycosylation reaction – proton transfer – BF_4_^–^ also plays an important role. BF_4_^–^ facilitates isopropyl group rotation by lowering the barrier from 28.4 (**TS2**) to 14.4 kcal/mol (**TS2’’**). At the same time, it inhibits proton transfer. Found **TS3’’** is 15.0 kcal/mol in contrast to other pathways where proton transfer occurs barrierless.

### Role of Activators in Glycosylation Reactions of Other Saccharides

It was experimentally shown by Ghorai *et al*.^9^ that TCA facilitates the reaction. However, the lowest barrier that they found for the reaction was 33.0 kcal/mol, which is too high for the reaction to proceed at 0 °C. Since their system is similar to ours (leaving group, catalyst), we tried to apply the gained knowledge to this system using alcohols in the previously found position as mediators. For modeling, the system used in the original paper was taken. We managed to decrease the reaction barrier to 21.6 kcal/mol (Figure S20). This agrees with the kinetic study by Ghorai *et al.* ^9^ that in the beginning of the reaction, glycosylation is slow and speeds up with the formation of TCA.

Addanki *et al.* ^8^ found barrier heights of 36.8 and 44.9 kcal/mol for their system, which is also similar to ours. For modeling, the system used in the original paper was taken. When the activators are placed the same way as in our system, the barrier height drops to 6.0 kcal/mol (Figure S21), which corresponds well to the experimental conditions (−50 °C).

## Conclusions

The goal of this study was to investigate the mechanism of a stereoselective glycosylation reaction in the presence of a pyridinium tetrafluoroborate catalyst using computational methods. It was found that the reaction is catalyzed only by BF_4_^–^ anion, and the cationic part does not play a significant role in catalysis. The substitution proceeds through an S_N_2 mechanism, rather than an S_N_1 mechanism. For S_N_2 substitution, several reaction paths, such as β-anomer formation, anchimeric participation of the acetoxy group, and the nucleophilic attack of the F^–^ anion from BF_4_^–^ to the anomeric carbon, were considered. Calculations showed that substitution occurred in one step, resulting in β-anomer formation. Herein, the H-bond donors such as *i*PrOH and TCA gave a significant impact, decreasing barrier height for S_N_2 substitution from 30.4 to 19.6 and 17.7 kcal/mol, respectively. We suggest that at the beginning of the reaction, *i*PrOH plays a more significant role as an activator, while toward the end of the reaction, the role of TCA becomes increasingly significant. In both cases, S_N_2 substitution is a rate-determining step for this reaction, followed by isopropanol group rotation and a proton transfer, which, depending on the mediator, can be barrierless or not. Our conclusions, based on computational studies of the reaction, are in good agreement with experimental data gathered by our laboratory and other research groups. Finally, we applied the obtained knowledge about activators to model the rate-determining glycosylation step in two similar reactions. Presence of activators decreases the calculated barrier from 33.0 to 21.6 kcal/mol and from 36.8 to 6.0 kcal/mol, which better agrees with stated reaction conditions.

## Experimental Procedures

### 2,3,4,6-Tetra-*O*-acetyl-α- **d** -galactopyranosyl Trichloroacetimidate Was Synthesized by a Known Procedure^51,52^ Starting from 1,2,3,4,6-penta-*O*-acetyl-β- **d** -galactopyranoside

Trichloroacetonitrile (0.55 mL, 5.5 mmol, 3.0 equiv) and K_2_CO_3_ (0.50 g, 3.7 mmol, 2.0 equiv) were added to a solution of 2,3,4,6-tetra-*O*-acetyl-d-galactopyranose (638 mg, 1.83 mmol, 1.0 equiv) in DCM (6 mL) at rt. The reaction was stirred overnight, and K_2_CO_3_ was separated by filtration through celite. The crude product was purified by silica gel flash-column chromatography (petroleum ether: EtOAc 3:1 → 2:1), yielding 2,3,4,6-tetra-*O*-acetyl-α-d-galactopyranosyl trichloroacetimidate (596 mg, 66%) as a white solid, ^1^H NMR (400 MHz, CDCl_3_) δ 8.66 (s, 1H), 6.60 (d, *J* = 3.4 Hz, 1H), 5.56 (dd, *J* = 3.1, 1.3 Hz, 1H), 5.43 (dd, *J* = 10.8, 3.1 Hz, 1H), 5.36 (dd, *J* = 10.9, 3.5 Hz, 1H), 4.48–4.39 (m, 1H), 4.17 (dd, *J* = 11.3, 6.6 Hz, 1H), 4.08 (dd, *J* = 11.3, 6.7 Hz, 1H), 2.17 (s, 3H), 2.03 (s, 3H), 2.02 (s, 3H), 2.01 (s, 3H); ^13^C­{^1^H} NMR (101 MHz, CDCl_3_) δ = 170.4, 170.2, 170.2, 170.1, 161.1, 93.7, 90.9, 69.1, 67.6, 67.5, 67.0, 61.4, 20.8, 20.8, 20.7, 20.7.

### 2,6-Di-*tert*-butylpyridinium Tetrafluoroborate (Catalyst **1**) was Synthesized by a Known Procedure^53^

One milliliter of acetyl chloride was added dropwise to 1 mL of dry methanol at 0 °C under an Ar atmosphere. After a few minutes, a solution of 2,6-di-*tert*-butylpyridine (200 mg, 1.05 mmol) in diethyl ether (0.5 mL) was added dropwise, and the reaction mixture was stirred at 0 °C under Ar for an additional 1 h. The solution was concentrated under reduced pressure to obtain a white solid. The solid was washed with diethyl ether (5 × 5 mL) to afford 2,6-di-*tert-*butylpyridinium chloride as a white solid (266 mg, quantitative yield), ^1^H NMR (400 MHz, CDCl_3_) δ 8.40 (tt, *J* = 8.2, 1.4 Hz, 1H), 7.76 (d, *J* = 8.1 Hz, 2H), 1.74 (s, 18H); ^13^C­{^1^H} NMR (101 MHz, CDCl_3_) δ = 166.2, 146.7, 121.7, 37.7, 29.8.

2,6-Di-*tert*-butylpyridinium chloride (200 mg, 0.878 mmol, 1.0 equiv) and sodium tetrafluoroborate (97 mg, 0.880 mmol, 1.0 equiv) were dissolved in 3 mL of DCM and stirred at 0 °C for 1 h. A white precipitate of sodium chloride was formed. Then, NaCl was filtered by washing with cold DCM and the solution was concentrated under reduced pressure to get 2,6-di-*tert-*butylpyridinium tetrafluoroborate as a white solid (204 mg, 69%), mp 195–197 °C, ^1^H NMR (400 MHz, CDCl_3_) δ 8.32 (t, *J* = 8.1 Hz, 1H), 7.73 (d, *J* = 8.1 Hz, 2H), 1.72 (s, 18H); ^13^C­{^1^H} NMR (101 MHz, CDCl_3_) δ = 166.1, 146.4, 121.7, 37.6, 29.6.

### Glycosylation Procedure

2,6-Di-*tert-*butylpyridinium tetrafluoroborate (2.8 mg, 0.01 mmol, 0.2 equiv) was weighed into a 4 mL screw-capped vial, 2,3,4,6-tetra-*O*-acetyl-α-d-galactopyranosyl trichloroacetimidate (24.6 mg, 0.05 mmol, 1.0 equiv), and CDCl_3_ (500 μL, 0.1 M solution of glycosyl donor, CDCl_3_ was dried over CaSO_4_) were added under an Ar atmosphere. The reaction mixture was stirred until all substances were dissolved, and then isopropanol (20 μL, 0.25 mmol, 5 equiv) as an acceptor was added. The reaction mixture was stirred at rt for 1 h (conversion 100%). The progress of the reaction was monitored by ^1^H NMR analysis. The reaction mixture was then concentrated under reduced pressure to obtain a crude gel-like product (24 mg, quantitative yield).

#### Isopropyl 2,3,4,6-tetra-*O*-acetyl-β- **d** -galactopyranoside^54^

^1^H NMR (400 MHz, CDCl_3_) δ 5.37 (dd, *J* = 3.5, 1.2 Hz, 1H), 5.16 (dd, *J* = 10.5, 7.9 Hz, 1H), 5.01 (dd, *J* = 10.5, 3.4 Hz, 1H), 4.50 (d, *J* = 8.0 Hz, 1H), 4.18 (dd, *J* = 11.2, 6.6 Hz, 1H), 4.11 (dd, *J* = 11.2, 6.9 Hz, 1H), 3.95–3.85 (m, 2H), 2.14 (s, 3H), 2.04 (s, 3H), 2.04 (s, 3H), 1.97 (s, 3H), 1.23 (d, *J* = 6.2 Hz, 3H), 1.14 (d, *J* = 6.1 Hz, 3H); ^13^C­{^1^H} NMR (101 MHz, CDCl_3_) δ = 170.6, 170.5, 170.4, 169.5, 100.4, 73.4, 71.2, 70.7, 69.2, 67.2, 61.5, 23.4, 22.2, 20.9, 20.8, 20.8, 20.8; HRMS (ESI): *m*/*z* calcd for C_17_H_26_O_10_+Na^+^: 413.1418 [M+Na]^+^; found: 413.1419.

## Computational Details

### Conformational Search

Conformational search was performed using the conformer-rotamer ensemble sampling tool (CREST) version 3.0 ^55^ and the Gaussian 16 rev C02 program package.^56^

CREST calculations were done in the gas phase using the GFNFF-xTB^11^ force field and followed by DFT calculations. To expand the ensemble, conformational searches using CREST were performed for both charged and neutral systems, and the correct system charges were accounted for at the DFT level of theory. The first Single Point (SP) energy calculations, further geometry optimization, and frequency calculations were performed in the gas phase using the B3LYP-D3­(BJ)/6-31+G* level of theory, and final Single Point (SP) energy calculations were done using B3LYP-D3­(BJ)/6-311++G** and the SMD solvent model to describe chloroform.

- Model 1 (Saccharide + catalyst (cation + anion)): the 188 conformers in the 6 kcal/mol energy window were selected for SP energy calculations. 66 conformers at 20 kcal/mol energy window were optimized with 40 cycles limit, after which 17 conformers remained in the 6.0 kcal/mol energy window, which were optimized to convergence. After removing the identical conformers, frequency and SP energy (with a larger basis set) calculations were performed for the 6 lowest energy conformers in the energy window of 4.0 kcal/mol.
- Model 2 (Saccharide + cation + *i*PrOH):
  Neutral CREST calculation: SP calculations were done for 1172 conformers, 508 conformers were optimized with 40 cycles limit, and 40 conformers to the convergence. Frequency and SP energy calculations were performed for the 3 lowest energy conformers in the energy window of 3.0 kcal/mol.
  Charged CREST calculation (with lowest energy conformer from neutral CREST calculation as a starting geometry): SP calculations were done for 17 conformers, all 17 conformers were optimized with 40 cycles limit, and 3 conformers to convergence. After removing duplicate geometries, frequency and SP energy calculations were performed for the 2 lowest energy conformers
- Model 3 (Saccharide + anion + *i*PrOH):
  - Neutral CREST calculation: SP calculations were done for 351 conformers, 141 conformers were optimized with 40 cycles limit, and 32 conformers – to convergence. Frequency and SP energy calculations were performed for the 10 lowest energy conformers in the energy window of 3.0 kcal/mol.
  - Charged CREST calculation (with lowest energy conformer from neutral CREST calculation as a starting geometry): SP calculations were done for 280 conformers, 169 conformers were optimized with 40 cycles limit, and 26 conformers – to the convergence. Frequency and SP energy calculations were performed for the 12 lowest energy conformers in the energy window of 3.0 kcal/mol.
- Finally, for all found conformers, the Boltzmann population at 298 K was calculated.

### Reaction Mechanism Study

All DFT calculations were performed with the Gaussian 16 rev C02 program package,^56^ and semiempirical calculations were performed using the xTB program.^57^

PES scans were performed to locate transition state (TS) geometries using either mPW1PW91-PFD/def2-SVP (DFT) or GFN2-xTB (semiempirical) methods.^57^

However, the TS structures obtained with semiempirical and DFT methods had different geometries and energies. Since DFT scans provided lower energy TSs, they were used for most TS searches. Guess geometries of TS and ground states were further optimized in the gas phase using mPW1PW91-PFD/def2-SVP level of theory, which showed good performance among five tested functionals (B3LYP^58^-D3­(BJ),^59^ PBE0-D3­(BJ), ωB97XD,^61^ M06-2X,^62^ and MPW1PW91-PFD^64^) and two basis sets (6-31+G­(2d,2p) and def2-SVP^65^), and were recommended by benchmark studies.^27–41^ The systems used for the performance test are shown in Figure S31, and the results of the performance test are shown in Table S13 and Figure S32. The calculations of the harmonic vibrational frequencies were performed to define the nature of the geometries found. All ground state geometries have zero imaginary frequencies, all transition state (TS) structures have 1 imaginary frequency corresponding to the making/breaking of the bond under investigation. Additionally, SP energies were calculated with SMD_(chloroform)_-mPW1PW91-PFD/def2-TZVPP^66^ method to get more accurate energy and take solvent effects into account.

Transition states were confirmed by IRC calculations, either LQA^67^ or EulerPC^68^ algorithms.

H-bond strengths were calculated using equations developed by Enamian *et al.* ^50^ based on electron density in critical points of corresponding bonds. Critical points, electron density, and potential energy density in them were calculated with Multiwfn 3.7 ^50,69,70^ software. In the case of neutral fragments, eq 1 was used and eq 2 was used for ions. eq 3 was also used for comparison since it is popular and has been frequently used for estimating H-bond strengths.^71,72^

y=223.08x+0.7423 1

y=−332.34x−1.0661 2

y=V(r)/2 3

where *x* is electron density, *V* is potential energy density, and *r* is bond radius.

### Visualization

Structures and calculation results were visualized using Visual Molecular Dynamics (VMD) 1.9.3,^73–75^ Molden 6.7,^76,77^ Avogadro 1.2.0,^78^ and Gaussview 6.1 ^79^ software packages.

## Supplementary Material





## Data Availability

The data underlying this study are available in the published article and its Supporting Information.

## Notes

Calculated energies and Boltzmann distribution of conformers, potential energy scans, NMR spectra (PDF)
Text file of all computed molecule Cartesian coordinates in a format for convenient visualization (XYZ)
