# Supplementary material for: Anion-Assisted Glycosylation of Galactose: A Computational Study
Source: J Org Chem. 2025 Dec 3;90(49):17507–17. doi: 10.1021/acs.joc.5c02404 (PMC12706777; doi:10.1021/acs.joc.5c02404)
Supplement: Supplementary file 1 [file jo5c02404_si_001.pdf]

Supporting information

Anion-assisted glycosylation of galactose: a computational study

Kerli Tali, Kaarel Erik Hunt, Kadri Kriis, Irina Osadchuk*, Tõnis Kanger*

Department of Chemistry and Biotechnology, Tallinn University of Technology,

Akadeemia tee 15, 12618 Tallinn, Estonia

Contents

General information S4

Table S1. Calculated energies and Boltzmann distribution of 4 lowest energy conformers found for the first model system (saccharide + catalyst (cation + anion)) S5

Table S2. Calculated energies and Boltzmann distribution of 3 lowest energy conformers found for the second model system (saccharide + cation + *i*PrOH) S5

Figure S1. Different H-bonds present in conformer structures. S5

Table S3. H-bond lengths and strengths in different conformers of model systems 2 and 3. To calculate the strength of hydrogen bonds, equation 1 is used for neutral fragments, and equation 2 is used for ions. H-bond strengths calculated S6

Table S4. Calculated energies and Boltzmann distribution of 10 lowest energy conformers for the third model system (saccharide + anion + *i*PrOH) S6

Figure S2. Potential energy scan for S_N1 substitution without addition of mediators.............S7

Figure S3. Potential energy scan for S_N1 substitution with addition of *i*PrOH at N. S8

Figure S4. Potential energy scan for S_N1 substitution with addition of *i*PrOH at O. S8

Figure S5. Potential energy scan for S_N1 substitution with addition of two *i*PrOHs. S9

Figure S6. Potential energy scan of F-glycosidic bond formation. S9

Table S5. Calculated energies for the first step of glycosylation reaction (substitution) *s*tructures without addition of activators...S10

Figure S7. 2-OAc dihedral angle rotation scan. S10

Table S6. Calculated energies for the first step of glycosylation reaction (substitution) structures activated by *i*PrOH...S11

Table S7. Calculated energies for the first step of glycosylation reaction (substitution) structures activated by TCA. S11

Figure S8. H-bonds in different structures. S12

Table S8. H-bond lengths and strengths of the substitution reagent, TSs and intermediate structures...S12

Table S9. H-bond lengths of substitution step reagent, TS and intermediate structures not included in Table S8. ....S13

Table S10. Electronic and Gibbs free energies of intermediate, TS and product structures for proton transfer. S15

Figure S9. H-bonds present in intermediates. S16

Table S11. H-bond strengths of intermediate and product with an *i*PrOH mediator. S16

Figure S10. Potential energy scan of proton transfer in presence of two *i*PrOH mediators and BF_4^- catalyst (system 1). S17

Figure S11. Geometries and energies of intermediates, TSs and product for glycosylation reaction occurring through TS1-4** (system 1). S17

Figure S12. Potential energy scan of proton transfer in presence of two TCA mediators (...S18

Figure S13. Geometries and energies of intermediates, TSs and product for glycosylation reaction occurring through TS1-4'' (system 3). S18

Table S12. H-bond strenghts of intermediates and products with TCA mediator. S19

Figure S14. Proton transfer in presence of two *i*PrOH mediators (system 2). S20

Figure S15. Proton transfer in presence of two TCA mediators (system 4). S21

Figure S16. Isopropyl group rotation for proton transfer in presence of two *i*PrOH mediators (system 2) S21

Figure S17. Structures of proton transfer system 2. ....S21

Figure S18. Isopropyl group rotation for proton transfer in presence of two TCA mediators (system 4) S22

Figure S20. Ghorai[1] structure stabilised by activator molecules. S23

Figure S21. Addanki[2] structure stabilised by activator molecules. S23

Figure S22. ^{1}H NMR spectrum of 2,3,4,6-tetra-O-acetyl-α-D-galactopyranosyl trichloroacetimidate S24

Figure S23. ^{13}C{1H} NMR spectrum of 2,3,4,6-tetra-O-acetyl-α-D-galactopyranosyl trichloroacetimidate S25

Figure S24. ^{1}H NMR spectrum of 2,6-di-tert-butylpyridinium chloride S26

Figure S25. ^{13}C{1H} NMR spectrum of 2,6-di-tert-butylpyridinium chloride S27

Figure S28. ^{1}H NMR spectrum of crude isopropyl 2,3,4,6-tetra-O-acetyl-β-D-galactopyranoside S30

Figure S29. ^{13}C{1H} NMR spectrum of crude isopropyl 2,3,4,6-tetra-O-acetyl-β-D-galactopyranoside S31

Figure S30. Glycosylation product formation in the crude reaction mixture monitored by ^{1}H NMR analysis (upper spectrum) compared to 2,3,4,6-tetra-O-acetyl-α-D-galactopyranosyl trichloroacetimidate (lower spectrum). S32

Figure S31. „Cationic β“ and „Anionic α“ pathways modelled to evaluate the performance of different DFT methods for the system under study. S33

Table S13. Gibbs free energies in kcal/mol calculated for „cationic β“ and „anionic α“ pathways using different methods. S33
Figure S32. a) Optimization and b) frequency calculation times for two different pathways with different methods. S34
Supplementary references..S34

General experimental information

Full assignment of ^{1}H and ^{13}C chemical shifts were based on the 1D and 2D (COSY, HSQC, HMBC) FT NMR spectra measured with a Bruker Avance III 400 MHz instrument. Residual solvent signals were used ($CDCl_3$: $\delta = 7.26$ ^{1}H NMR, 77.2 $^{13}C\{^{1}H\}$ NMR) as internal standards. High-resolution mass spectra were recorded with an Agilent Technologies 6540 UHD Accurate-Mass QTOF LC/MS spectrometer by using AJS-ESI ionisation. Prior to analysis the instrument was calibrated in the mass range of m/z 50–3200. Melting points were determined using polarising optical microscope Nagema-K8. Precoated Merck silica gel 60 F254 plates were used for TLC (thin layer chromatography) and column chromatography was performed with Merck 60 (0.040–0.063 mm) mesh silica gel. Commercial reagents, and solvents were generally used as received. DCM, ethyl acetate (EtOAc) and acetone were distilled over phosphorus pentoxide, MeOH and toluene over sodium. Petroleum ether (PE) had a boiling point of 40–60 °C.

Table S1. Calculated energies and Boltzmann distribution of 4 lowest energy conformers found for the first model system (saccharide + catalyst (cation + anion))

Geometry	Electronic Energy, Hartrees B3LYP-D3BJ/6-31+G*	Thermal Correction to Gibbs Free Energy, Hartrees	Single point calculations, B3LYP-D3(BJ)/6-311++G**	Relative Gibbs free energy, kcal/mol	Boltzmann distr., %
**1 (A)**	-3797.5077272	0.618314	-3798.2702768	0.00	64.8
**2 (B)**	-3797.5016141	0.617910	-3798.2692760	0.37	34.4
**C**	-3797.5015609	0.619875	-3798.2675955	2.66	0.7
**D**	-3797.5078321	0.622520	-3798.2684242	3.80	0.1

Table S2. Calculated energies and Boltzmann distribution of 3 lowest energy conformers found for the second model system (saccharide + cation + *i*PrOH)

Geometry	Electronic Energy, Hartrees B3LYP-D3BJ/6-31+G*	Thermal Correction to Gibbs Free Energy, Hartrees	Single point calculations, B3LYP-D3(BJ)/6-311++G**	Relative Gibbs free energy, kcal/mol	Boltzmann distr., %
**3 (A)**	-3567.2259648	0.715184	-3567.9528634	0.00	57.7
**4 (B)**	-3567.2223474	0.715514	-3567.9527246	0.29	35.3
**C**	-3567.2225575	0.714010	-3567.9493315	1.48	4.8
**D**	-3567.21021806	0.712996	-3567.9476042	1.92	2.2

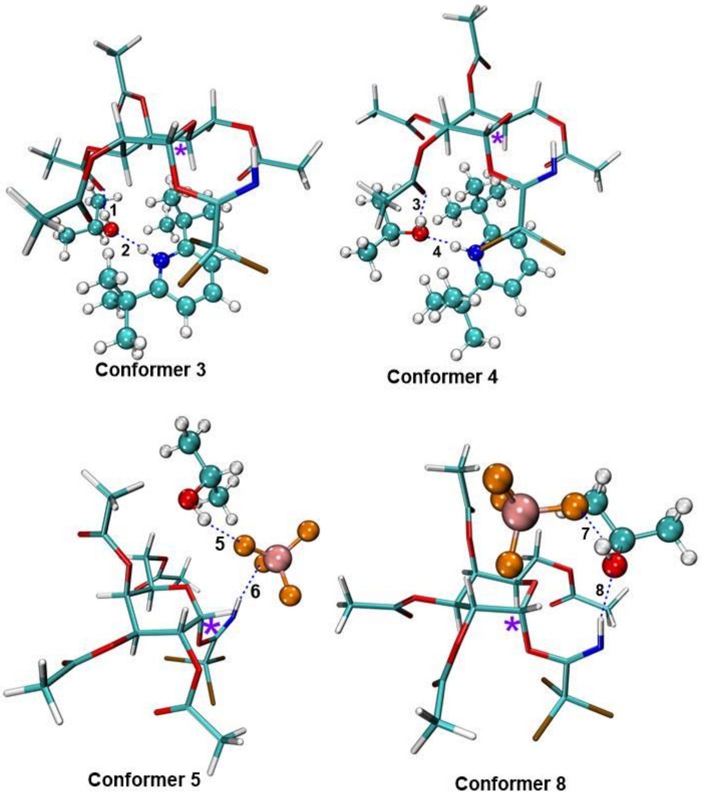

Figure S1. Different H-bonds present in conformer structures. The numbers denote an H-bond, which are marked in blue.

Table S3. H-bond lengths and strengths in different conformers of model systems 2 and 3. To calculate the strength of hydrogen bonds, equation 1 is used for neutral fragments, and equation 2 is used for ions. H-bond strengths calculated with frequently used equation 3 is also provided for comparison.

Conformer	Bond	H-bond length, Å	Electron density, a. u.	Potential energy density (eV)	H-bond strengths, kcal/mol		
					Eq. 1	Eq. 2	Eq. 3
**Conf. 3**	1	1.78	0.03561341216	-0.03412980741	-	12.9	11.7
	2	1.87	0.03035302326	-0.02588395546	7.5	-	9.5
**Conf. 4**	3	1.84	0.03009584084	-0.02766146218	11.2	-	9.5
	4	1.88	0.03035746655	-0.02584406218	11.1	-	9.4
**Conf. 5**	5	1.83	0.02809300432	-0.02667019709	-	10.4	8.8
	6	1.99	0.01881266072	-0.01508801404	-	7.3	5.9
**Conf. 6**	5	1.82	0.02876966390	-0.02731386102	-	10.6	9.0
**Conf. 7**	5	1.80	0.03050969989	-0.02975524815	-	11.2	9.6
**Conf. 8**	7	1.76	0.03354677076	-0.03340736727	-	12.2	10.5
	8	1.90	0.02904942990	-0.02472595790	7.2	-	9.1
**Conf. 9**	5	1.85	0.02727935173	-0.02545573021	-	10.1	8.6
**Conf. 10**	5	1.83	0.02763823677	-0.02632739513	-	10.3	8.7
	6	2.12	0.01356428804	-0.009845131945	-	5.8	4.3

Table S4. Calculated energies and Boltzmann distribution of 10 lowest energy conformers for the third model system (saccharide + anion + *i*PrOH)

Geometry	Electronic Energy, Hartrees B3LYP-D3BJ/6-31+G*	Thermal Correction to Gibbs Free Energy, Hartrees	Single point calculations, B3LYP-D3(BJ)/6-311++G**	Relative Gibbs free energy, kcal/mol	Boltzmann distr., %
**5 (A)**	-3428.5082562	0.406616	-3429.2103098	0.00	25.9
**6 (B)**	-3428.5052811	0.405858	-3429.2092972	0.16	19.8
**7 (C)**	-3428.5060222	0.405651	-3429.2090770	0.17	19.4
**8 (D)**	-3428.5067628	0.407498	-3429.2103805	0.51	10.9
**9 (E)**	-3428.5050788	0.405958	-3429.2084757	0.74	7.4
**10 (F)**	-3428.5012647	0.404951	-3429.2073694	0.80	6.7
**G**	-3428.5058302	0.409252	-3429.2111733	1.11	4.0
**H**	-3428.5054924	0.407082	-3429.2089631	1.14	3.8
**I**	-3428.5082867	0.408758	-3429.2093898	1.92	1.0
**J**	-3428.5059484	0.408231	-3429.2084546	2.18	0.7
**K**	-3428.5055947	0.408858	-3429.2087821	2.36	0.4

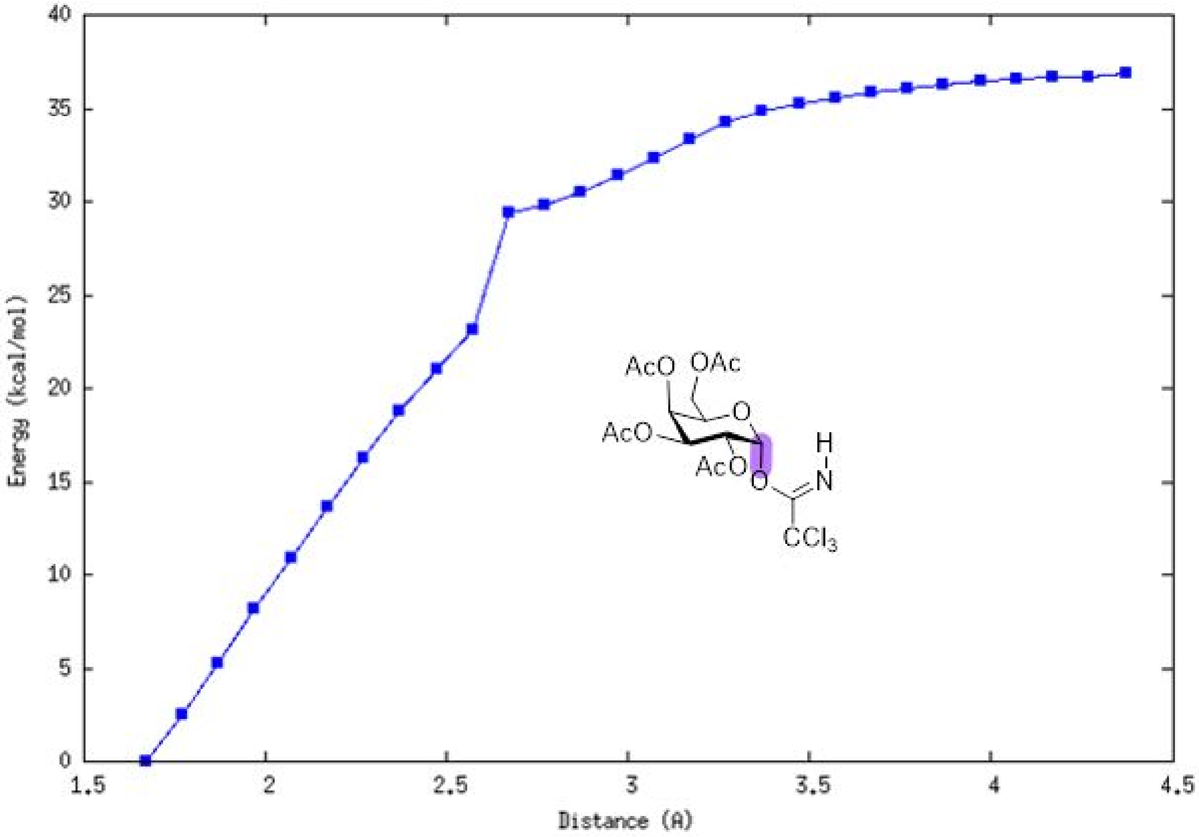

Figure S2. Potential energy scan for S_N1 substitution without addition of mediators. The bond under investigation is marked in purple.

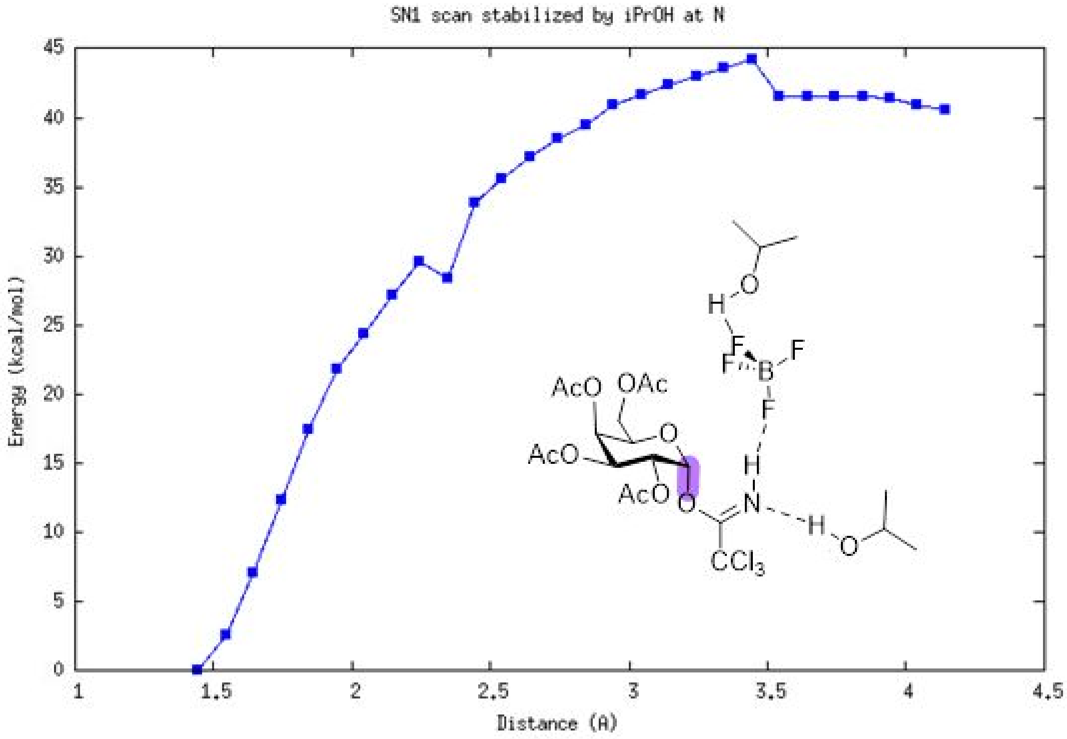

Figure S3. Potential energy scan for S_N1 substitution with addition of *i*PrOH at N. The bond under investigation is marked in purple.

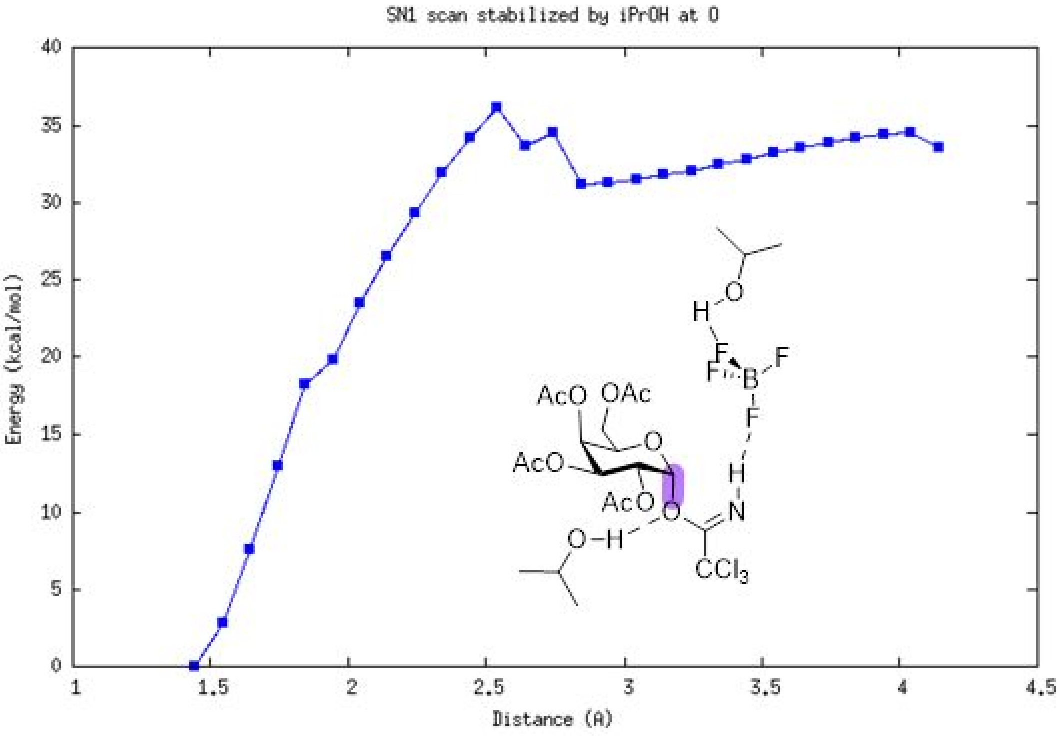

Figure S4. Potential energy scan for S_N1 substitution with addition of *i*PrOH at O. The bond under investigation is marked in purple.

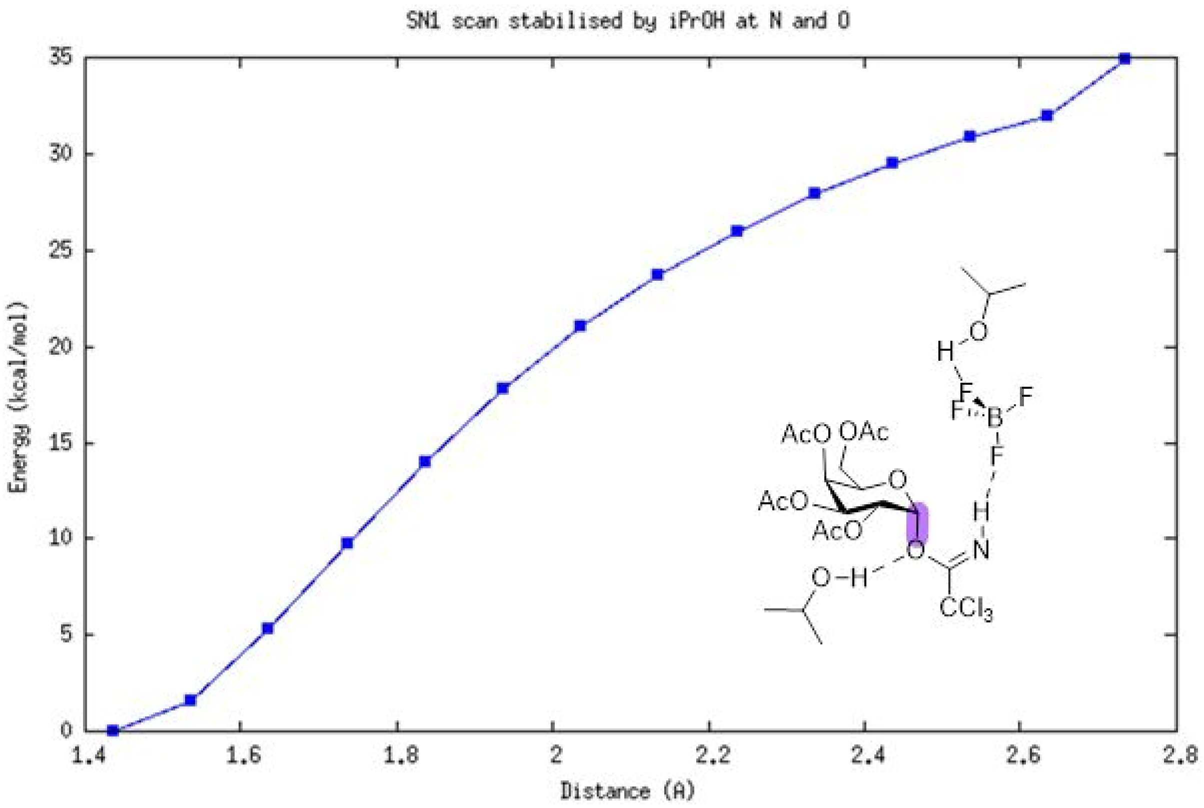

Figure S5. Potential energy scan for S_N1 substitution with addition of two *i*PrOHs. The bond under investigation is marked in purple.

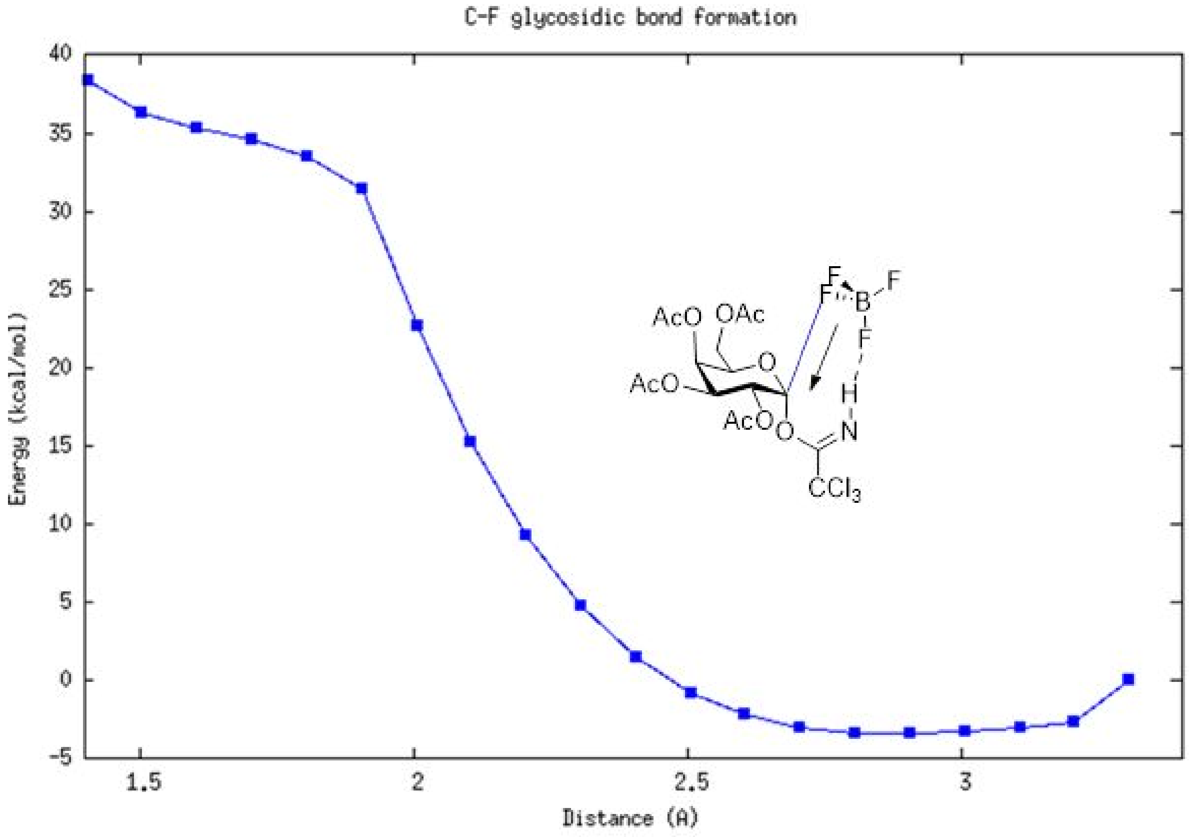

Figure S6. Potential energy scan of F-glycosidic bond formation.

Table S5. Calculated energies for the first step of glycosylation reaction (substitution) structures without addition of activators.

Geometry	Anion	Thermal Correction to Gibbs Free Energy, Hartrees	Single point calculations, B3LYP-D3(BJ)/6-311++G**	Relative energy, kcal/mol
**Reagent**	BF_4^-	0.406616	-3428.5082562	0.0
**TS1-1**	BF_4^-	0.416570	-3428.8443157	34.1
**TS1-2**	BF_4^-	0.416176	-3428.8515754	29.3
**TS1-3**	BF_4^-	0.417098	-3428.8432615	35.1
**TS1-4**	BF_4^-	0.417385	-3428.8508705	30.5
**INT1-2**	BF_4^-	0.414095	-3428.8673520	18.1
**INT1-4**	BF_4^-	0.418744	-3428.8618500	24.5

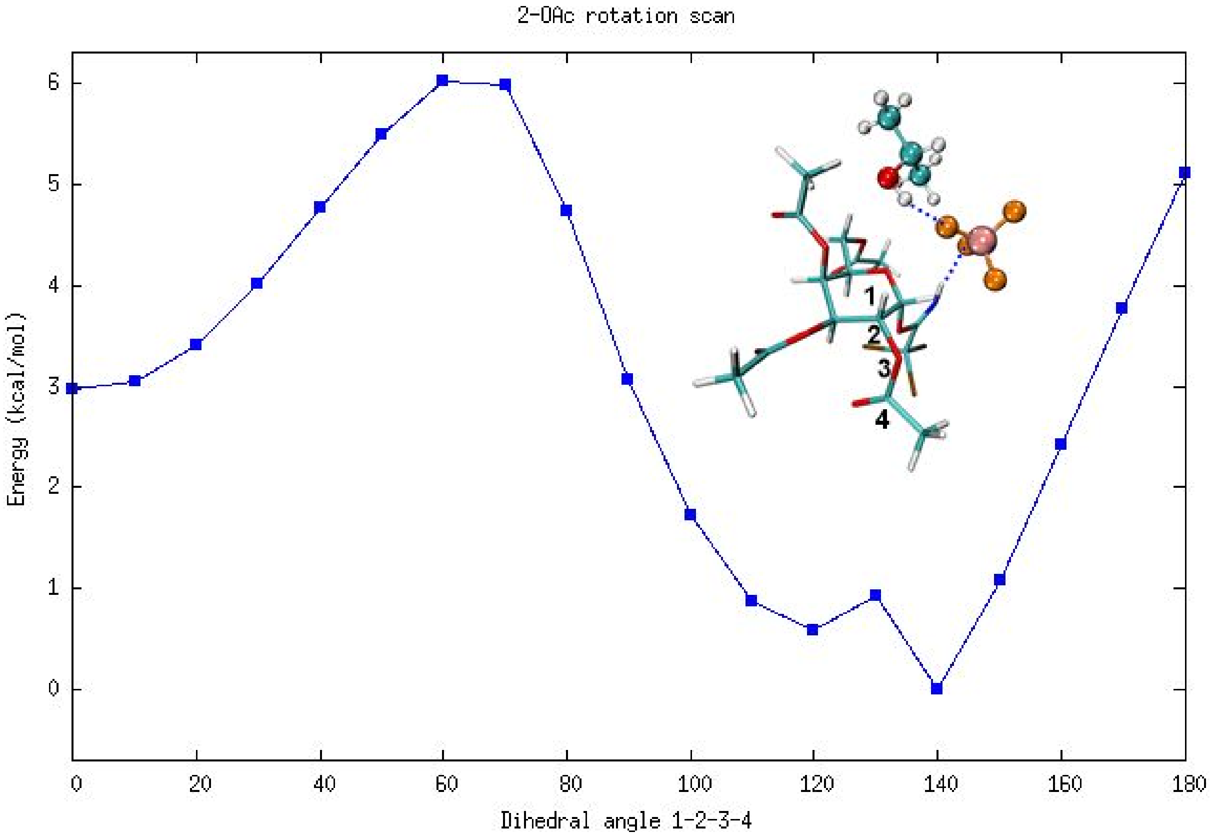

Figure S7. 2-OAc dihedral angle rotation scan. Dihedral angle 1-2-3-4 changed.

Table S6. Calculated energies for the first step of glycosylation reaction (substitution) structures activated by *i*PrOH.

Geometry	Anion	Thermal Correction to Gibbs Free Energy, Hartrees	Single point calculations, B3LYP-D3(BJ)/6-311++G**	Relative energy, kcal/mol
**Reagent***	BF_4^-	0.519871	-3620.2264286	0.0
**Reagent****	BF_4^-	0.622563	-3817.7298778	0.0
**Reagent****	-	0.612988	-3390.2649628	0.0
**TS1-2* (A)**	BF_4^-	0.520383	-3623.2740041	27.5
**TS1-2*(B)**	BF_4^-	0.516801	-3623.2727984	26.0
**TS1-2****	BF_4^-	0.621066	-3817.6927219	22.4
**TS1-2****	NO	0.612959	-3393.3042868	25.2
**TS1-4* (A)**	BF_4^-	0.521524	-3623.2723835	26.7
**TS1-4*(B)**	BF_4^-	0.518642	-3623.2759201	25.2
**TS1-4****	BF_4^-	0.623108	-3817.6986338	19.6
**TS1-4****	-	0.614491	-3392.9786748	25.2
**INT1-2*(A)**	BF_4^-	0.517194	-3623.2897268	15.6
**INT1-2*(B)**	BF_4^-	0.515172	-3623.2890299	14.8
**INT1-2****	BF_4^-	0.623146	-3817.7042500	19.2
**INT1-4*(A)**	BF_4^-	0.520718	-3623.2859166	17.7
**INT1-4*(B)**	BF_4^-	0.520903	-3623.2868814	19.7
**INT1-4****	BF_4^-	0.620779	-3817.7105330	11.0

Table S7. Calculated energies for the first step of glycosylation reaction (substitution) structures activated by TCA.

Geometry	Anion	Thermal Correction to Gibbs Free Energy, Hartrees	Single point calculations, B3LYP-D3(BJ)/6-311++G**	Relative energy, kcal/mol
**Reagent’**	BF_4^-	0.456312	-5013.4829490	0.0
**Reagent’’**	BF_4^-	0.491565	-6605.2184859	0.0
**Reagent’’**	-	0.480269	-6176.7596056	0.0
**TS1-2’(A)**	BF_4^-	0.451927	-5017.0205527	24.2
**TS1-2’(B)**	BF_4^-	0.451296	-5017.0170383	25.3
**TS1-2’’**	BF_4^-	0.486901	-6605.1827183	19.5
**TS1-4’(A)**	BF_4^-	0.450119	-5017.0152208	25.8
**TS1-4’(B)**	BF_4^-	0.449569	-5017.0190020	23.0
**TS1-4’’**	BF_4^-	0.484515	-6605.1681441	17.7
**INT1-2’(A)**	BF_4^-	0.451927	-5017.0374924	12.1
**INT1-2’(B)**	BF_4^-	0.447782	-5017.0322326	15.9
**INT1-2’’**	BF_4^-	0.488253	-6605.1995101	2.5
**INT1-4’(A)**	BF_4^-	0.450027	-5017.0290578	17.1
**INT1-4’(B)**	BF_4^-	0.453575	-5017.0332207	16.6
**INT1-4’’**	BF_4^-	0.483076	-6605.2032520	-5.6
**Reagent*’**	BF_4^-	0.555772	-5207.6775806	0.0
**TS1-2*’**	BF_4^-	0.554451	-5211.4328818	24.6
**TS1-4*’**	BF_4^-	0.553355	-5211.4410458	18.8
**INT1-2*’**	BF_4^-	0.556821	-5211.4543874	18.4
**INT1-4*’**	BF_4^-	0.553444	-5211.4683342	3.3
**INT1-4*’**	BF_4^-	0.554533	-5211.4567504	11.2

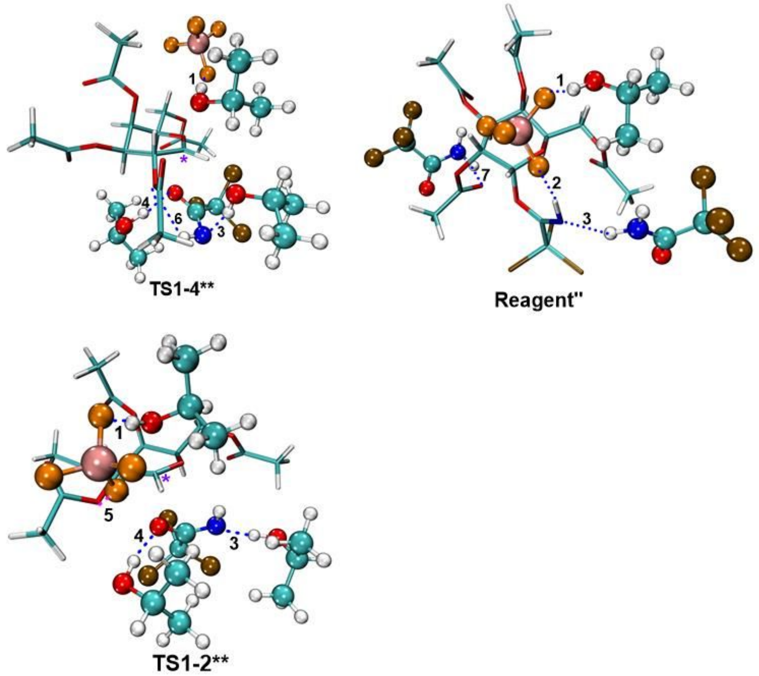

Figure S8. H-bonds in different structures. The numbers denote an H-bond, which are marked in blue.

Table S8. H-bond lengths and strengths of the substitution reagent, TSs and intermediate structures. To calculate the strength of hydrogen bonds, equation 1 is used for neutral fragments, and equation 2 is used for ions. H-bond strengths calculated with frequently used equation 3 is also provided for comparison.

**Structure**	**H-bond**	**H-bond length, Å**	**Electron density, a. u.**	**Potential energy density (eV)**	**H-bond strengths, kcal/mol**		
					**Eq. 1**	**Eq. 2**	**Eq. 3**
**TS1-2**	1	1.50	0.06711993973	-0.08086177013	-	23.4	21.1
**INT1-2**	1	1.26	0.05702455784	-0.06611278158	-	20.0	17.9
**TS1-4**	1	1.59	0.05251222376	-0.05998771394	-	18.5	16.5
**INT1-4**	1	1.36	0.09748993936	-0.1310898706	-	33.5	30.6
**Reagent****	1	1.74	0.03527651309	-0.03635369921	-	12.8	11.1
	2	1.93	0.02255040891	-0.01958055024	-	8.6	7.1
	3	1.89	0.03315355703	-0.02664000432	8.1	-	10.4
	4	1.88	0.02572305516	-0.02326761413	6.4	-	8.1
**TS1-2****	1	1.60	0.05159107082	-0.05841812293	-	18.2	16.2
	3	1.74	0.04935564137	-0.04407264843	11.8	-	15.5
	4	1.80	0.03441419379	-0.03223921622	8.4	-	10.8
**INT1-2****	1	1.32	0.1102264217	-0.1558338539	-	37.7	34.6
	3	1.70	0.05384375060	-0.04906707589	12.8	-	16.9
	4	1.71	0.04309589089	-0.04311929389	10.4	-	13.5
**TS1-4****	1	1.64	0.04629704812	-0.05125820433	-	16.5	14.5
	3	1.84	0.03769707304	-0.03171541471	9.2	-	11.8
	4	1.75	0.04244837292	-0.04131519737	10.2	-	13.3
	6	2.58	0.007570121020	-0.004464777738	2.4	-	2.4
**INT1-4****	1	1.34	0.09684503017	-0.1314170187	-	33.3	30.4
	3	1.64	0.05998144445	-0.05343635337	14.1	-	18.8
	4	1.73	0.04385937730	-0.03523041779	10.5	-	13.8
	6	2.44	0.01152649001	-0.009035183365	3.3	-	3.6
**Reagent’’**	1	1.74	0.03498344898	-0.03634549457	-	12.7	11.0

	2	2.02	0.01715916332	-0.01374374520	-	6.8	5.4
	3	1.89	0.03396466535	-0.02665205054	8.3	-	10.7
	7	1.88	0.02756114605	-0.02400035593	6.9	-	8.6
**TS1-2’’**	1	1.61	0.1177814468	-0.1723140609	-	40.2	37.0
	3	1.67	0.06968399350	-0.06526512051	16.3	-	21.9
	4	1.66	0.04845257351	-0.04869564061	11.6	-	15.2
**INT1-2’’**	1	1.29	0.1177814468	-0.1723140609	-	40.2	37.0
	3	1.60	0.06968399350	-0.06526512051	16.3	-	21.9
	4	1.66	0.04845257351	-0.04869564061	11.6	-	15.2
**TS1-4’’**	1	1.61	0.05038979922	-0.05729691052	-	17.8	15.8
	3	1.73	0.05095368107	-0.04425178423	12.1	-	16.0
	4	1.76	0.03918698350	-0.03686584540	9.5	-	12.3
	6	2.64	0.006065131005	-0.003303824378	2.1	-	1.9
**INT1-4’’**	1	1.30	0.1137404947	-0.1637606011	-	38.9	35.7
	3	1.67	0.05944898814	-0.05352630534	14.0	-	18.7
	4	1.81	0.03456839689	-0.03199620327	8.5	-	10.8
	6	2.60	0.006979411206	-0.003809200329	2.3	-	2.2
	7	1.88	0.02768040360	-0.02436781004	6.9	-	8.7

Table S9. H-bond lengths of substitution step reagent, TS and intermediate structures not included in Table S8.

**Structure**	**H-bond**	**Bond length, Å**
**Reagent***	1	1.83
	2	1.85
	3	1.85
**TS1-2*(A)**	1	1.54
	4	1.81
**INT1-2*(A)**	1	1.42
	4	1.78
**Reagent***	1	1.83
	2	1.85
	3	1.85
**TS1-2*(B)**	1	1.57
	3	1.71
**INT1-2*(B)**	1	1.24
	3	1.74
**Reagent’**	1	1.79
	7	1.93
**TS1-2’(A)**	1	1.55
	4	1.98
**INT1-2’(A)**	1	1.23
	4	1.73
**Reagent’**	1	1.79
	7	1.93
**TS1-2’(B)**	1	1.54
	3	1.64
**INT1-2’(B)**	1	1.31
	2	2.27
	3	1.56
**Reagent*’**	1	1.73
	2	1.92
	3	1.88
	7	1.78
**TS1-2*’**	1	1.63
	2	2.33

	3	1.67
	4	1.92
**INT1-2*’**	1	1.28
	3	1.59
	4	1.83
**Reagent***	1	1.83
	2	1.85
	3	1.85
**TS1-4*(A)**	1	1.60
	4	1.74
	6	3.04
**INT1-4*(A)**	1	1.33
	4	1.67
	6	2.62
**Reagent***	1	1.83
	2	1.85
	3	1.85
**TS1-4*(B)**	1	1.36
	3	1.61
	6	2.36
**INT1-4*(B)**	1	1.62
	3	1.79
	6	2.45
**Reagent’**	1	1.79
	7	1.93
**TS1-4’(A)**	1	1.62
	4	1.74
	6	2.86
**INT1-4’(A)**	1	1.35
	4	1.65
	6	2.92
**Reagent’**	1	1.79
	7	1.93
**TS1-4’(B)**	1	1.58
	3	1.71
	6	2.67
**INT1-4’(B)**	1	1.33
	3	1.64
	6	2.49
**Reagent*’**	1	1.73
	2	1.92
	3	1.88
	7	1.78
**TS1-4A*’**	1	1.62
	3	1.74
	4	1.78
	6	2.67
**INT1-4A*’**	1	1.33
	3	1.67
	4	1.72
	6	2.56
**Reagent*’**	1	1.73
	2	1.92
	3	1.88
	7	1.78
**TS1-4B*’**	1	1.60
	3	1.84
	4	1.71

**TS1-1**	1	1.59
**TS1-3**	1	1.53
	2	2.52

Table S10. Electronic and Gibbs free energies of intermediate, TS and product structures for proton transfer.

**Geometry**	**Anion**	**Thermal Correction to Gibbs Free Energy, Hartrees**	**Single point calculations, B3LYP-D3(BJ)/6-311++G****	**Relative Gibbs free energy, kcal/mol**
**INT1****	BF_4^-	0.620779	-3817.7105325	11.0
**INT2****	BF_4^-	0.626195	-3817.7106557	14.3
**TS2****	BF_4^-	0.625541	-3817.6969005	22.5
**INT2****	BF_4^-	0.622885	-3817.7086301	13.5
**Product****	BF_4^-	0.622097	-3817.7679943	-24.2
**INT1''**	BF_4^-	0.492602	-6605.2044513	9.4
**TS2''**	BF_4^-	0.487971	-6605.1918320	14.4
**INT2''**	BF_4^-	0.490044	-6605.2056293	7.1
**INT3''**	BF_4^-	0.491508	-6605.2028147	9.8
**TS3''**	BF_4^-	0.492644	-6605.1955655	15.0
**Product''**	BF_4^-	0.493344	-6605.2603117	-25.2
**INT1****	-	0.616185	-3392.982211	25.0
**TS2****	-	0.621572	-3392.982169	28.4
**Product****	-	0.61588	-3393.066505	-28.1
**INT1''**	-	0.48396	-6180.472823	23.2
**TS2''**	-	0.48232	-6180.469014	24.6
**Product''**	-	0.482125	-6180.536925	-24.6

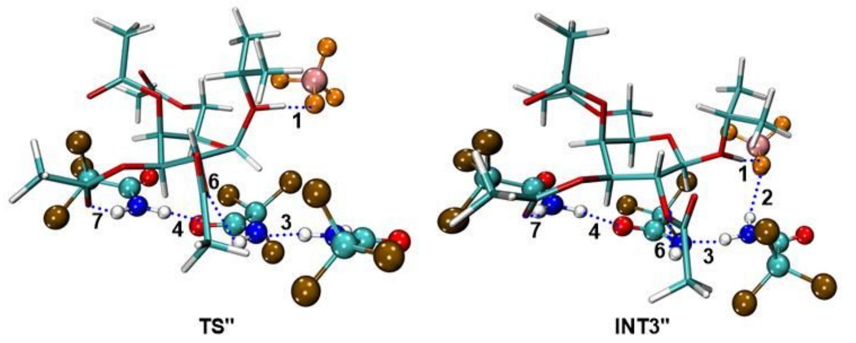

Figure S9. H-bonds present in intermediates.

Table S11. H-bond strengths of intermediate and product with an *i*PrOH mediator. To calculate the strength of hydrogen bonds, equation 1 is used for neutral fragments, and equation 2 is used for ions. H-bond strengths calculated with frequently used equation 3 is also provided for comparison.

**Structure**	**H-bond**	**H-bond length, Å**	**Electron density, a. u.**	**Potential energy density (eV)**	**H-bond strengths, kcal/mol**		
					**Eq. 1**	**Eq. 2**	**Eq. 3**
**Reagent****	1	1.74	0.03527651309	-0.03635369921	-	12.8	11.1
	2	1.93	0.02255040891	-0.01958055024	-	8.6	7.1
	3	1.89	0.03315355703	-0.02664000432	8.1	-	10.4
	4	1.88	0.02572305516	-0.02326761413	6.4	-	8.1
**TS1-2****	1	1.60	0.05159107082	-0.05841812293	-	18.2	16.2
	3	1.74	0.04935564137	-0.04407264843	11.8	-	15.5
	4	1.80	0.03441419379	-0.03223921622	8.4	-	10.8
**INT1-2****	1	1.32	0.1102264217	-0.1558338539	-	37.7	34.6
	3	1.70	0.05384375060	-0.04906707589	12.8	-	16.9
	4	1.71	0.04309589089	-0.04311929389	10.4	-	13.5
**TS1-4****	1	1.64	0.04629704812	-0.05125820433	-	16.5	14.5
	3	1.84	0.03769707304	-0.03171541471	9.2	-	11.8
	4	1.75	0.04244837292	-0.04131519737	10.2	-	13.3
	6	2.58	0.007570121020	-0.004464777738	2.4	-	2.4
**INT1-4****	1	1.34	0.09684503017	-0.1314170187	-	33.3	30.4
	3	1.64	0.05998144445	-0.05343635337	14.1	-	18.8
	4	1.73	0.04385937730	-0.03523041779	10.5	-	13.8
**INT2****	1	1.33	0.1064216061	-0.1484295603	-	36.4	33.4
	3	1.67	0.05741631241	-0.05347153445	13.6	-	18.0
	4	1.69	0.04922928132	-0.04907419367	11.7	-	15.4
**TS2****	1	1.28	0.1218538134	-0.1820117760	-	41.6	38.2
	3	1.74	0.04800656251	-0.04259732783	11.5	-	15.1
	4	1.66	0.05351677966	-0.05448032514	12.7	-	16.8
**INT3****	1	1.30	0.1148796814	-0.1663619784	-	39.2	36.0
	3	1.71	0.05106041783	-0.04606625708	12.1	-	16.0
	4	1.63	0.05652950030	-0.05851243023	13.4	-	17.7
**Product****	1	1.80	0.03113475665	-0.03020911539	-	11.4	9.8
	3	1.76	0.04214471986	-0.03990635572	10.1	-	13.2
	4	1.75	0.04215171092	-0.04081058078	10.1	-	13.2
	6	1.97	0.02524098756	-0.02083835747	6.4	-	7.9

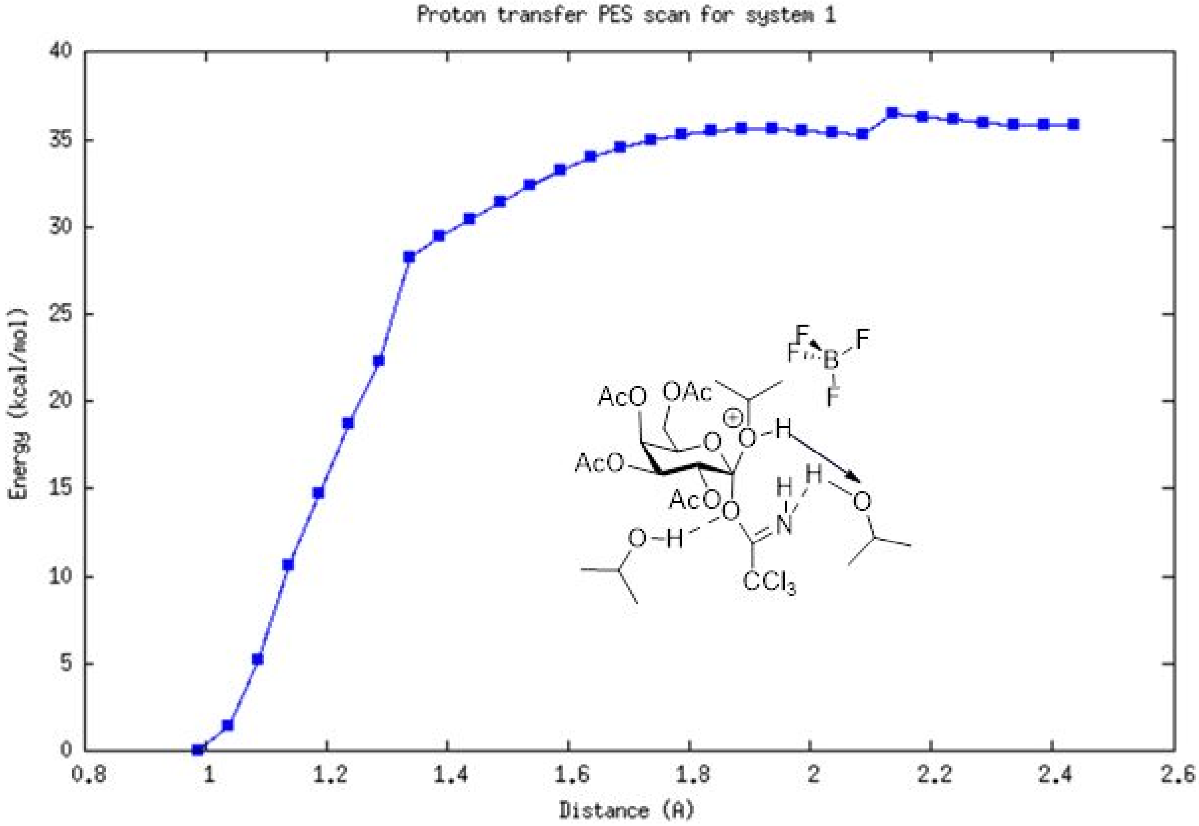

Figure S10. Potential energy scan of proton transfer in presence of two *i*PrOH mediators and BF_4^- catalyst (system 1).

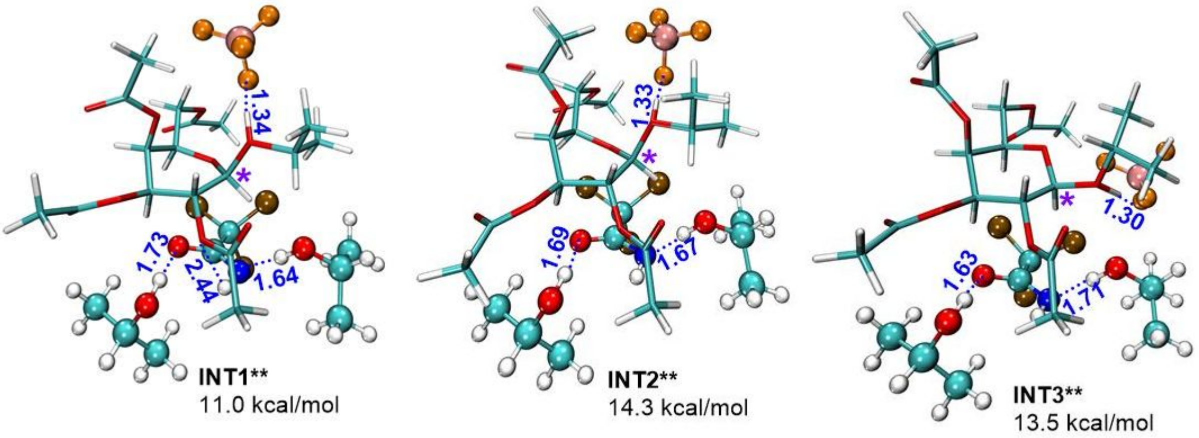

Figure S11. Geometries and energies of intermediates, TSs and product for glycosylation reaction occurring through TS1-4 (system 1).** Anomeric carbon is marked with a purple asterisk. H-bonds are marked in blue. Distances are given in Å. H-bond strengths are given in **Figure S11** and **Table S12**.

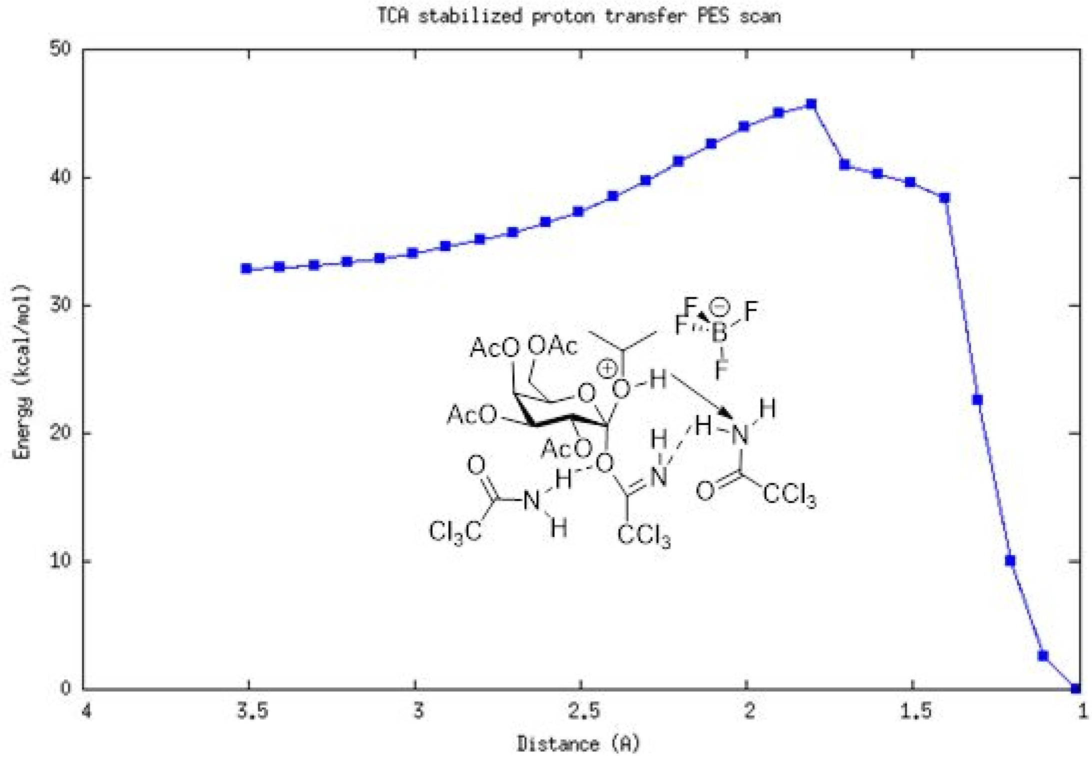

Figure S12. Potential energy scan of proton transfer in presence of two TCA mediators (system 3).

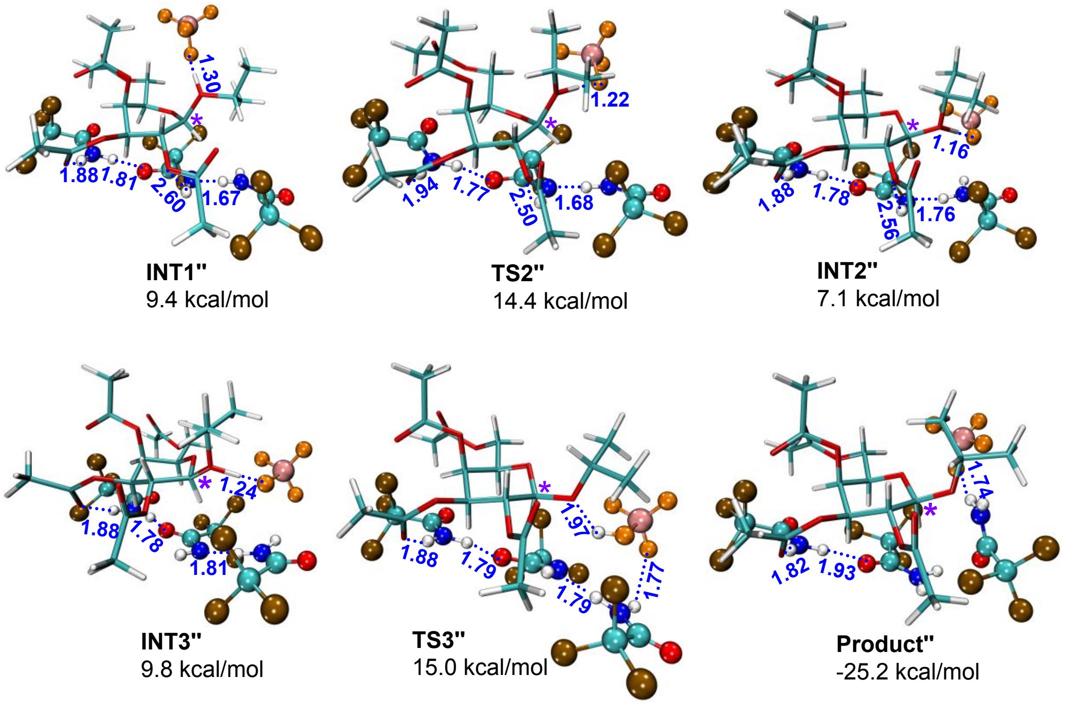

Figure S13. Geometries and energies of intermediates, TSs and product for glycosylation reaction occurring through TS1-4'' (system 3). Anomeric carbon is marked with a purple asterisk. H-bonds are marked in blue. Distances are given in Å. H-bond strengths are given in **Figure S11** and **Table S12**.

Table S12. H-bond strenghts of intermediates and products with TCA mediator. To calculate the strength of hydrogen bonds, equation 1 is used for neutral fragments, and equation 2 is used for ions. H-bond strengths calculated with frequently used equation 3 is also provided for comparison.

Structure	H-bond	Bond length	Electron density	Potential energy density (V)	H-bond strengths, kcal/mol		
					Eq. 1	Eq. 2	Eq. 3
**Reagent''**	1	1.74	0.03498344898	-0.03634549457	-	12.7	11.0
	2	2.02	0.01715916332	-0.01374374520	-	6.8	5.4
	3	1.89	0.03396466535	-0.02665205054	8.3	-	10.7
	7	1.88	0.02756114605	-0.02400035593	6.9	-	8.6
**TS1-2''**	1	1.61	0.1177814468	-0.1723140609	-	40.2	37.0
	3	1.67	0.06968399350	-0.06526512051	16.3	-	21.9
	4	1.66	0.04845257351	-0.04869564061	11.6	-	15.2
**INT1-2''**	1	1.29	0.1177814468	-0.1723140609	-	40.2	37.0
	3	1.60	0.06968399350	-0.06526512051	16.3	-	21.9
	4	1.66	0.04845257351	-0.04869564061	11.6	-	15.2
**TS1-4''**	1	1.61	0.05038979922	-0.05729691052	-	17.8	15.8
	3	1.73	0.05095368107	-0.04425178423	12.1	-	16.0
	4	1.76	0.03918698350	-0.03686584540	9.5	-	12.3
	6	2.64	0.006065131005	-0.003303824378	2.1	-	1.9
**INT1-4''**	1	1.30	0.1137404947	-0.1637606011	-	38.9	35.7
	3	1.67	0.05944898814	-0.05352630534	14.0	-	18.7
	4	1.81	0.03456839689	-0.03199620327	8.5	-	10.8
	6	2.60	0.006979411206	-0.003809200329	2.3	-	2.2
	7	1.88	0.02768040360	-0.02436781004	6.9	-	8.7
**TS2''**	1	1.22	0.1423122122	-0.2322552698	-	48.4	44.7
	3	1.68	0.05757807778	-0.05136590428	13.6	-	18.1
	4	1.77	0.03820421210	-0.03642683015	9.3	-	12.0
	6	2.50	0.008457381238	-0.005038840547	2.6	-	2.7
	7	1.94	0.02424239776	-0.02040908421	6.2	-	7.6
**INT2-1''**	1	1.16	0.1686560774	-0.3117890454	-	57.1	52.9
	3	1.76	0.04723198076	-0.03996286551	11.3	-	14.8
	4	1.78	0.03736122720	-0.03550376778	9.1	-	11.7
	6	2.56	0.007135895844	-0.003981399161	2.3	-	2.2
	7	1.88	0.02736880785	-0.02412529638	6.8	-	8.6
**INT2-2''**	1	1.24	0.1319395718	-0.2066044126	-	44.9	41.4
	3	1.81	0.04241344638	-0.03467419888	10.2	-	13.3
	4	1.78	0.03795139619	-0.03586926009	9.2	-	11.9
	7	1.88	0.02759106238	-0.02441527948	6.9	10.2	8.7
	11	1.96	0.02035857784	-0.01683919837	-	7.8	6.4
**TS3''**	1	1.97	0.02171093527	-0.01782631473	-	8.3	6.8
	2	1.77	0.03368617804	-0.03292873909	-	12.3	10.6
	3	1.79	0.04440807953	-0.03657667138	10.6	-	13.9
	4	1.79	0.03647725956	-0.03404207063	8.9	-	11.4
	7	1.88	0.02740293203	-0.02418383274	6.9	-	8.6
**Product''**	2	1.74	0.03611610390	-0.03621871392	-	13.1	11.3
	4	1.93	0.02589209055	-0.02183898684	6.5	-	8.1
	7	1.82	0.03190528834	-0.02957129950	-	11.7	10.0

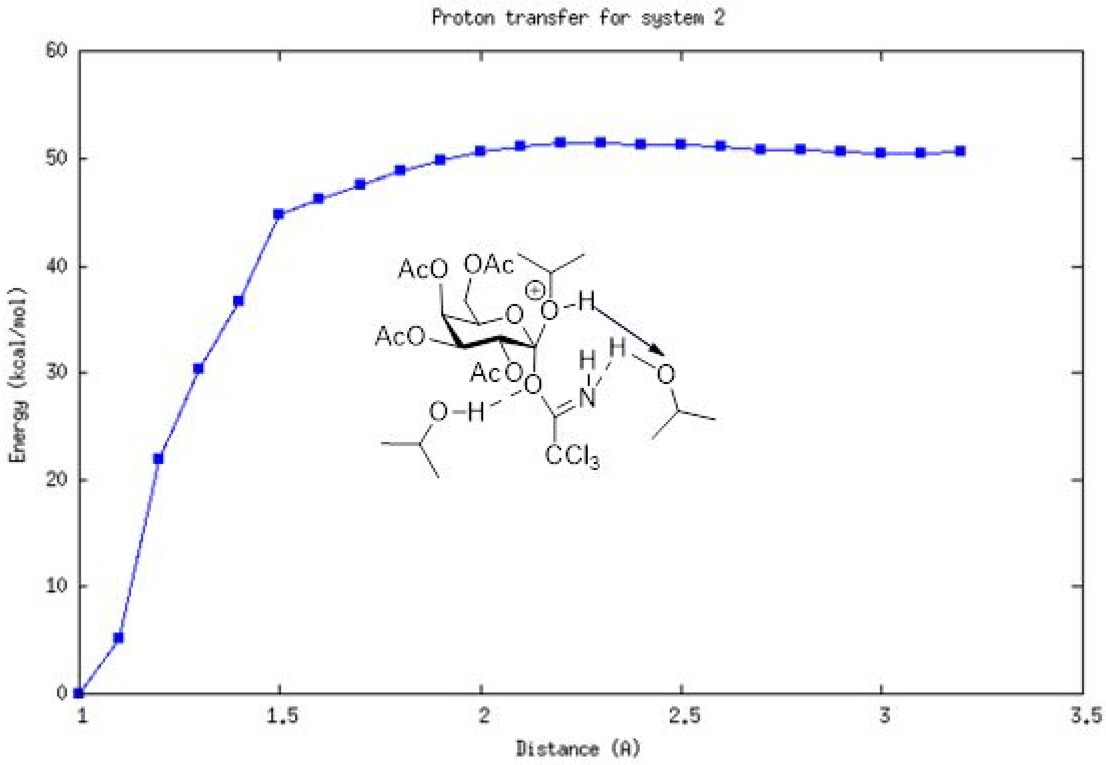

Figure S14. Proton transfer in presence of two *i*PrOH mediators (system 2).

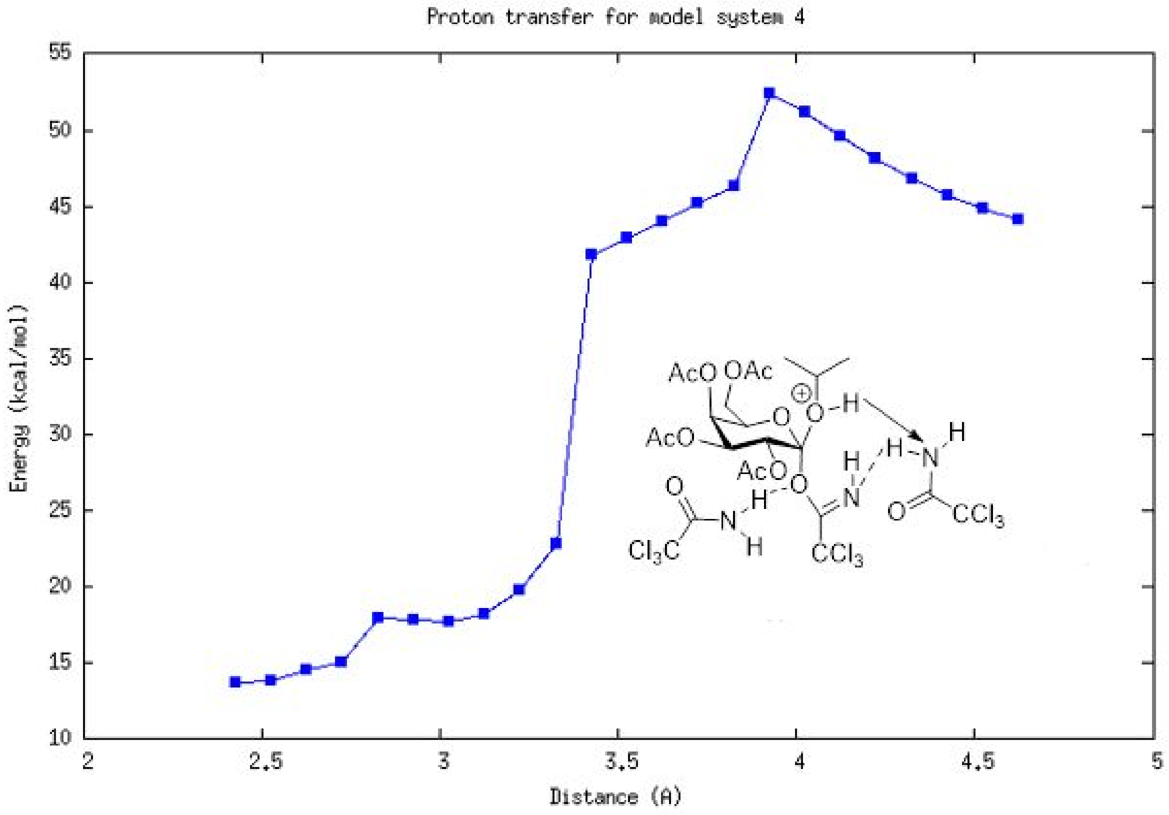

Figure S15. Proton transfer in presence of two TCA mediators (system 4).

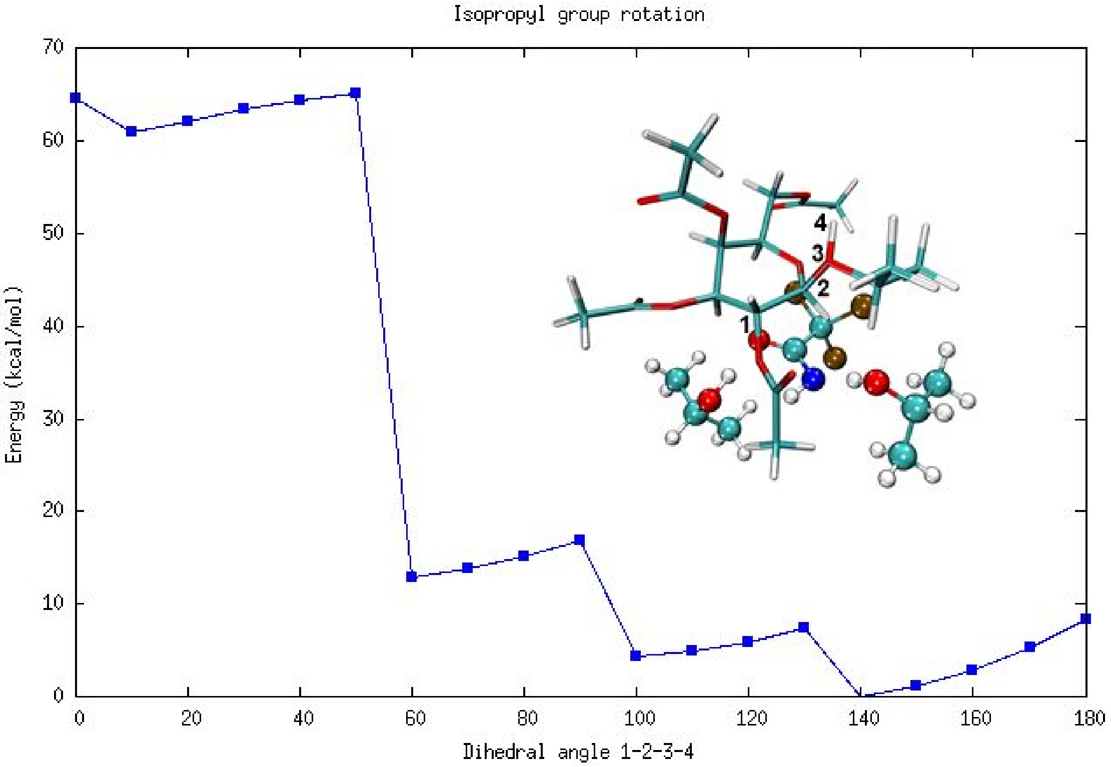

Figure S16. Isopropyl group rotation for proton transfer in presence of two *i*PrOH mediators (system 2).

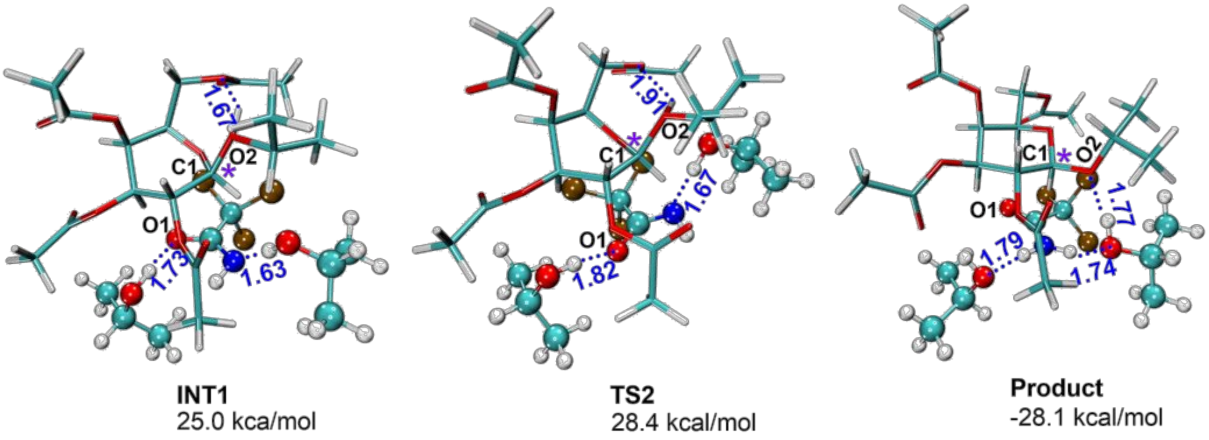

Figure S17. Structures of proton transfer system 2.

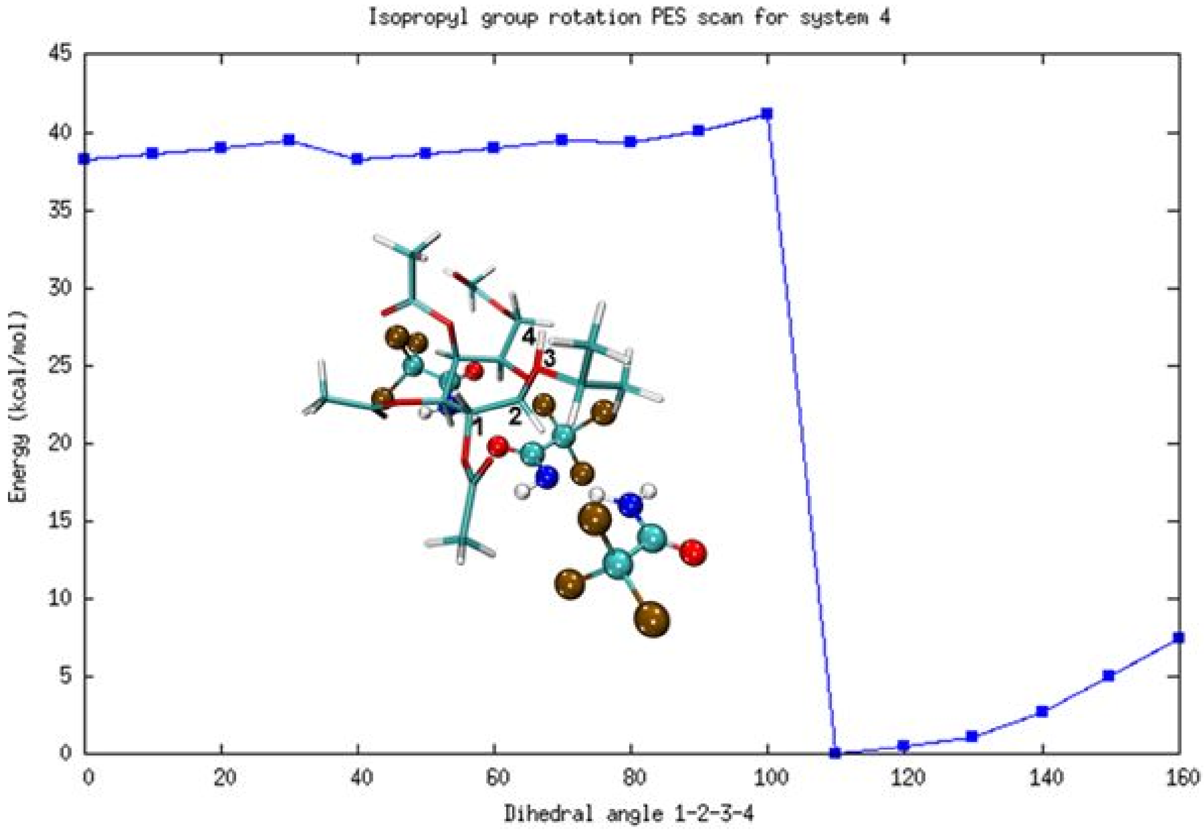

Figure S18. Isopropyl group rotation for proton transfer in presence of two TCA mediators (system 4).

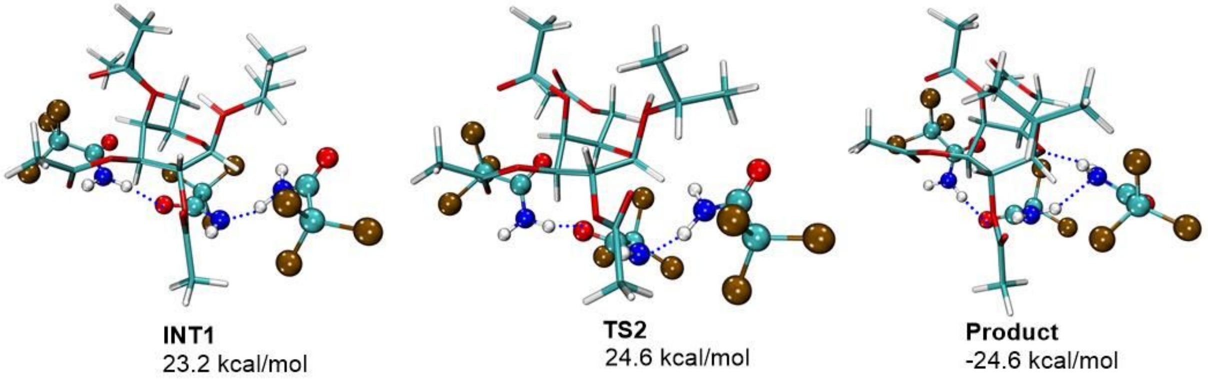

Figure S19. Structures of intermediates and products for proton transfer system 4.

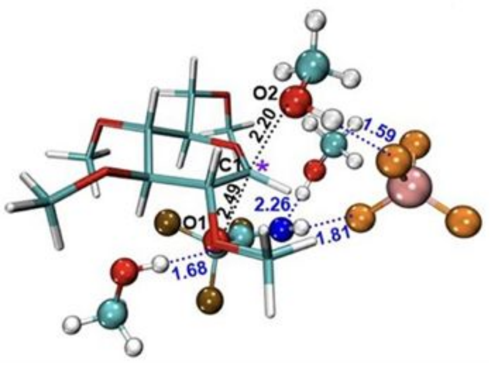

Figure S20. Ghorai[1] structure stabilised by activator molecules.

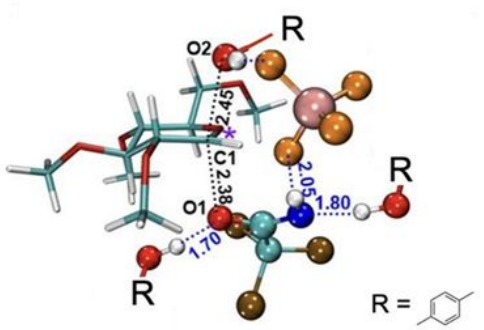

Figure S21. Addanki[2] structure stabilised by activator molecules.

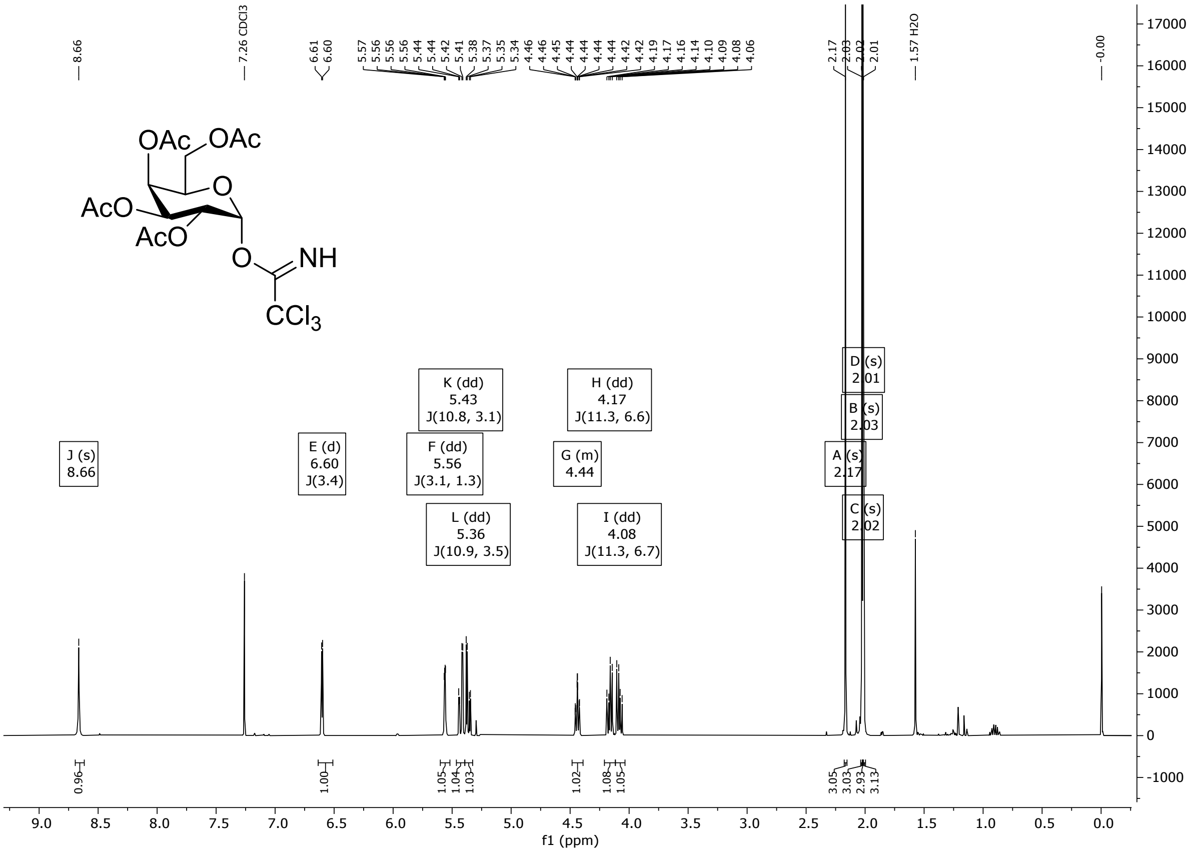

Figure S22. ^{1}H NMR spectrum of 2,3,4,6-tetra-*O*-acetyl-α-D-galactopyranosyl trichloroacetimidate (400 MHz, $CDCl_3$).

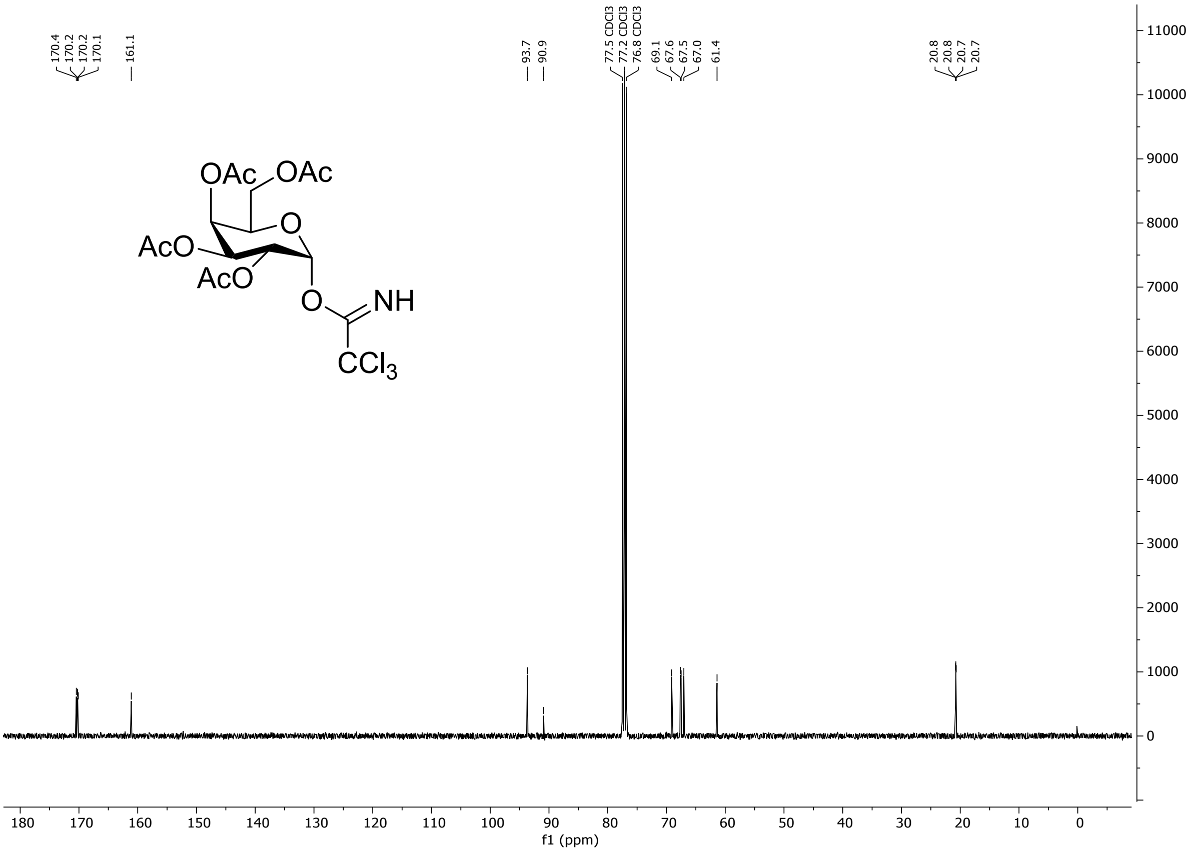

Figure S23. $^{13}C\{^{1}H\}$ **NMR spectrum of 2,3,4,6-tetra-*O*-acetyl-α-D-galactopyranosyl trichloroacetimidate** (101 MHz, $CDCl_3$).

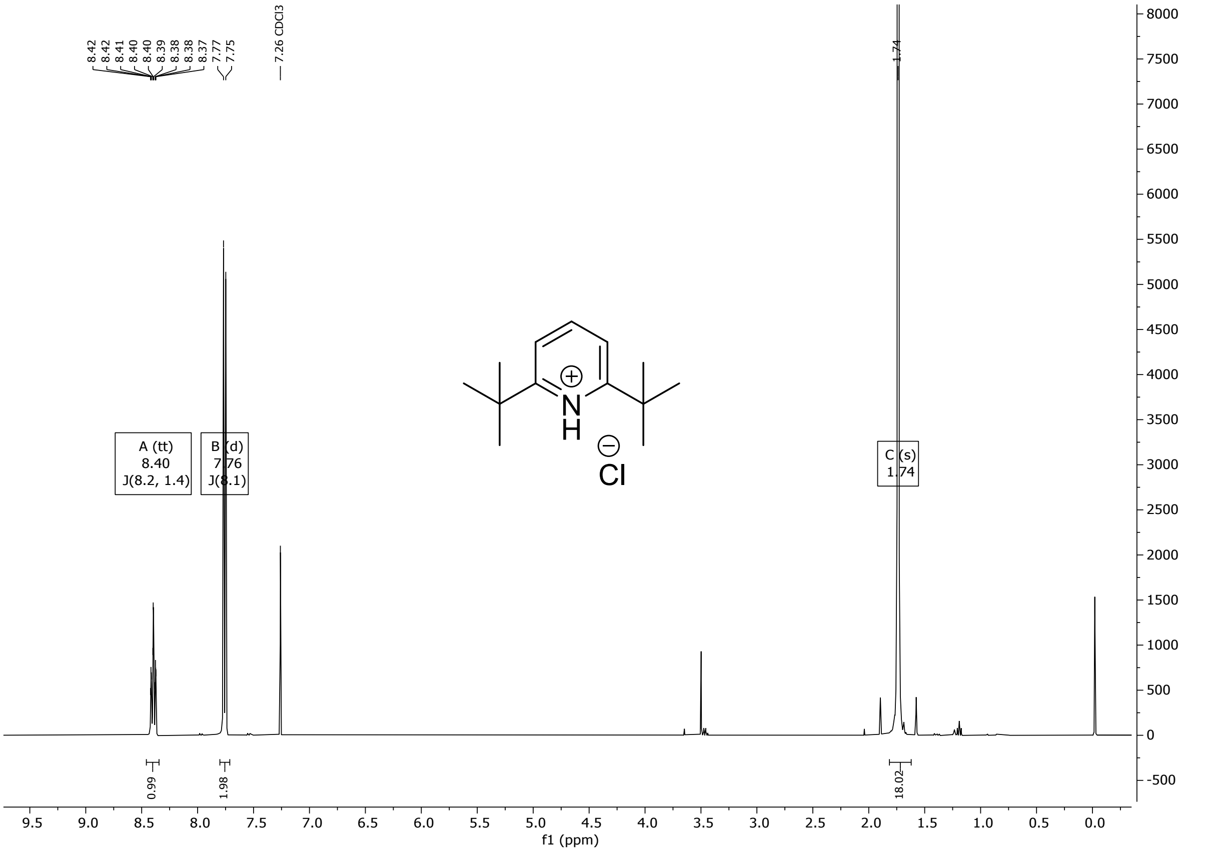

Figure S24. ^{1}H NMR spectrum of 2,6-di-*tert*-butylpyridinium chloride (400 MHz, $CDCl_3$).

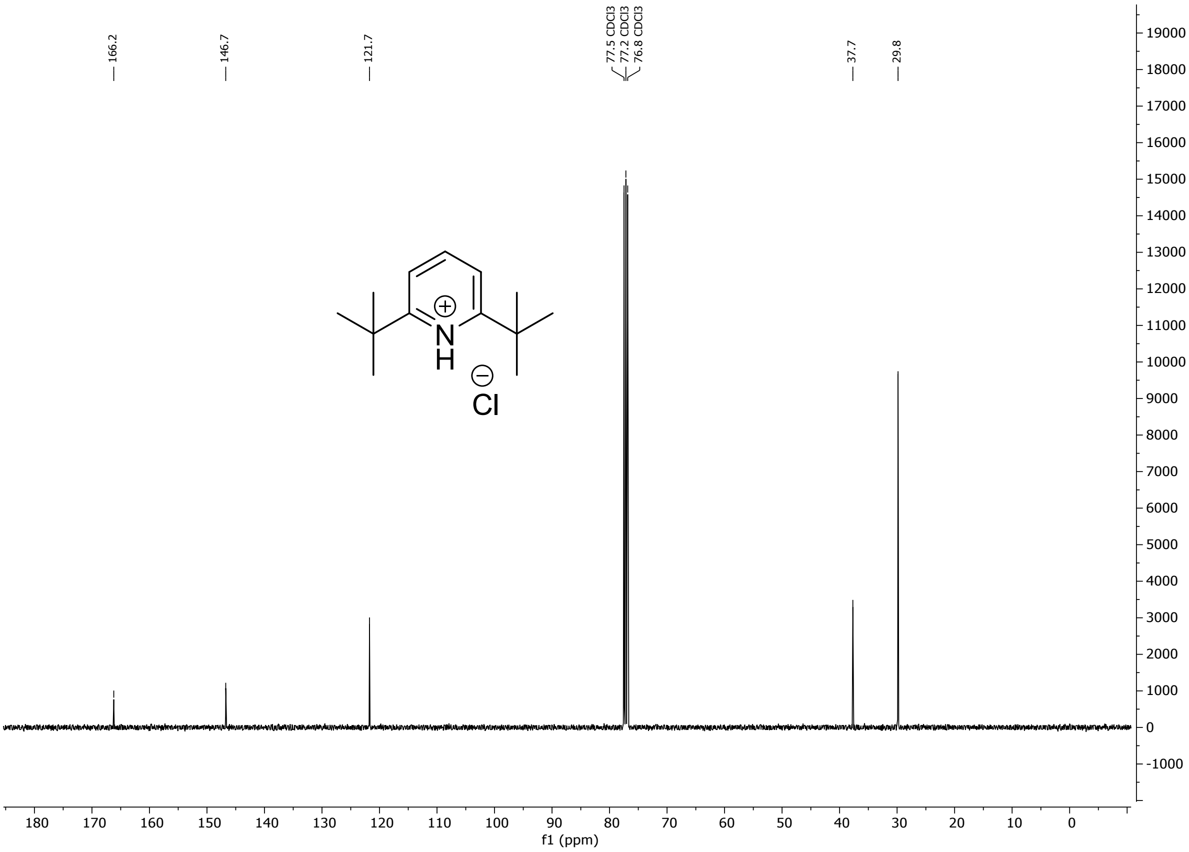

Figure S25. ^{13}**C{**^{1}**H} NMR spectrum of 2,6-di-*tert*-butylpyridinium chloride** (101 MHz, $CDCl_3$).

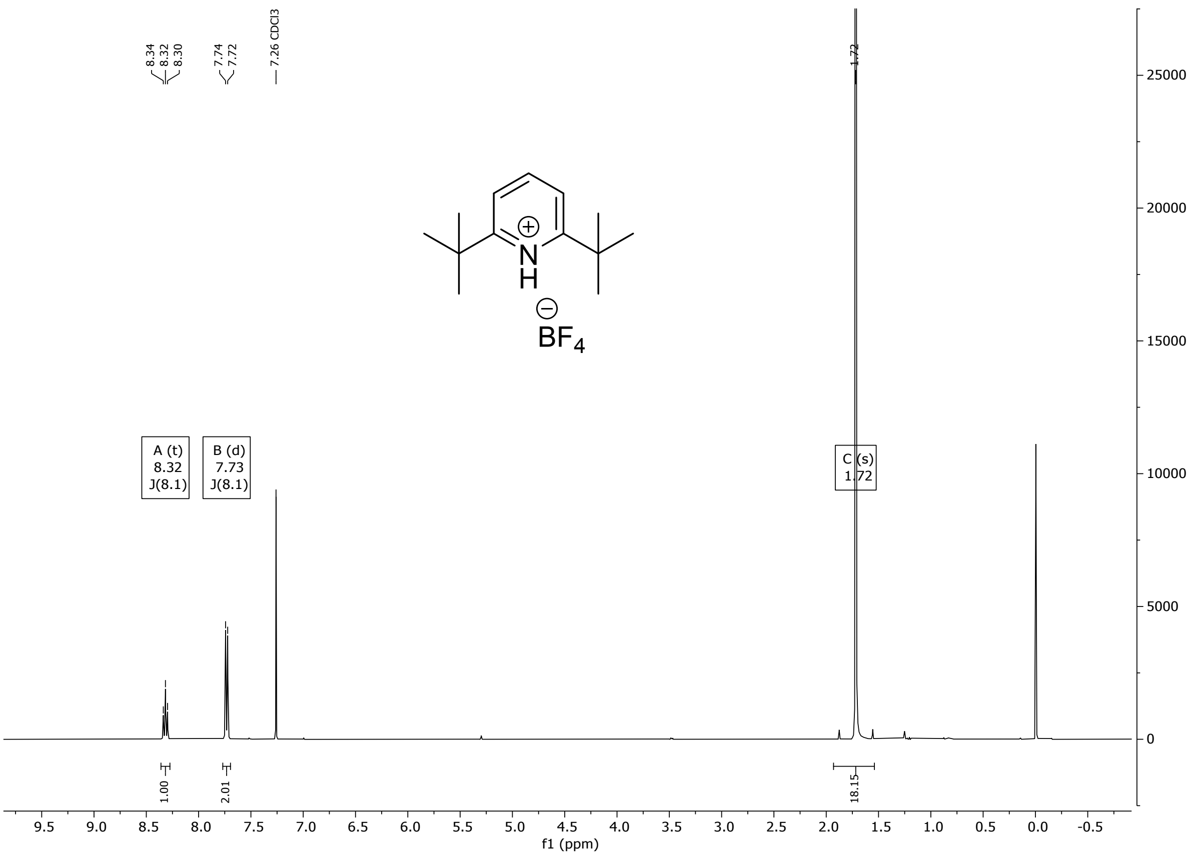

Figure S26. ^{1}H NMR spectrum of 2,6-di-*tert*-butylpyridinium tetrafluoroborate (400 MHz, $CDCl_3$).

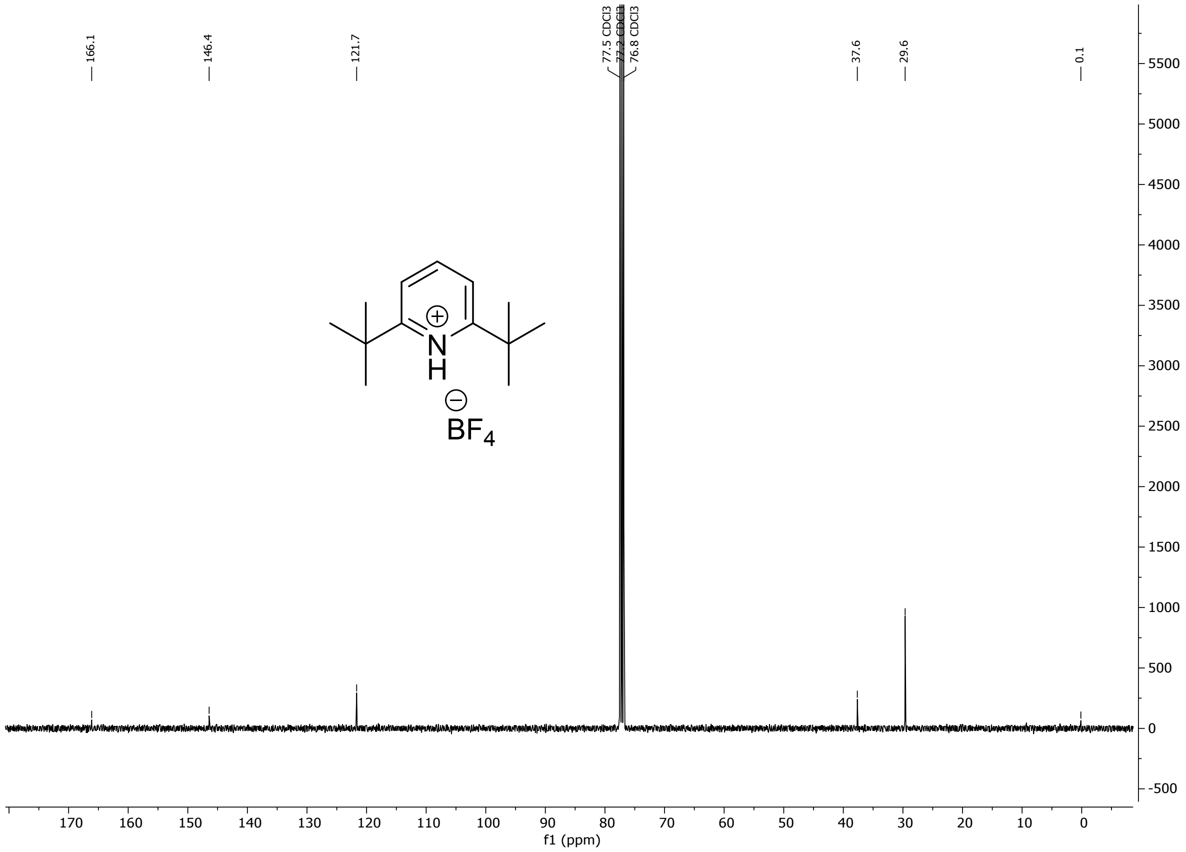

Figure S27. $^{13}C\{^{1}H\}$ NMR spectrum of 2,6-di-*tert*-butylpyridinium tetrafluoroborate (101 MHz, $CDCl_3$).

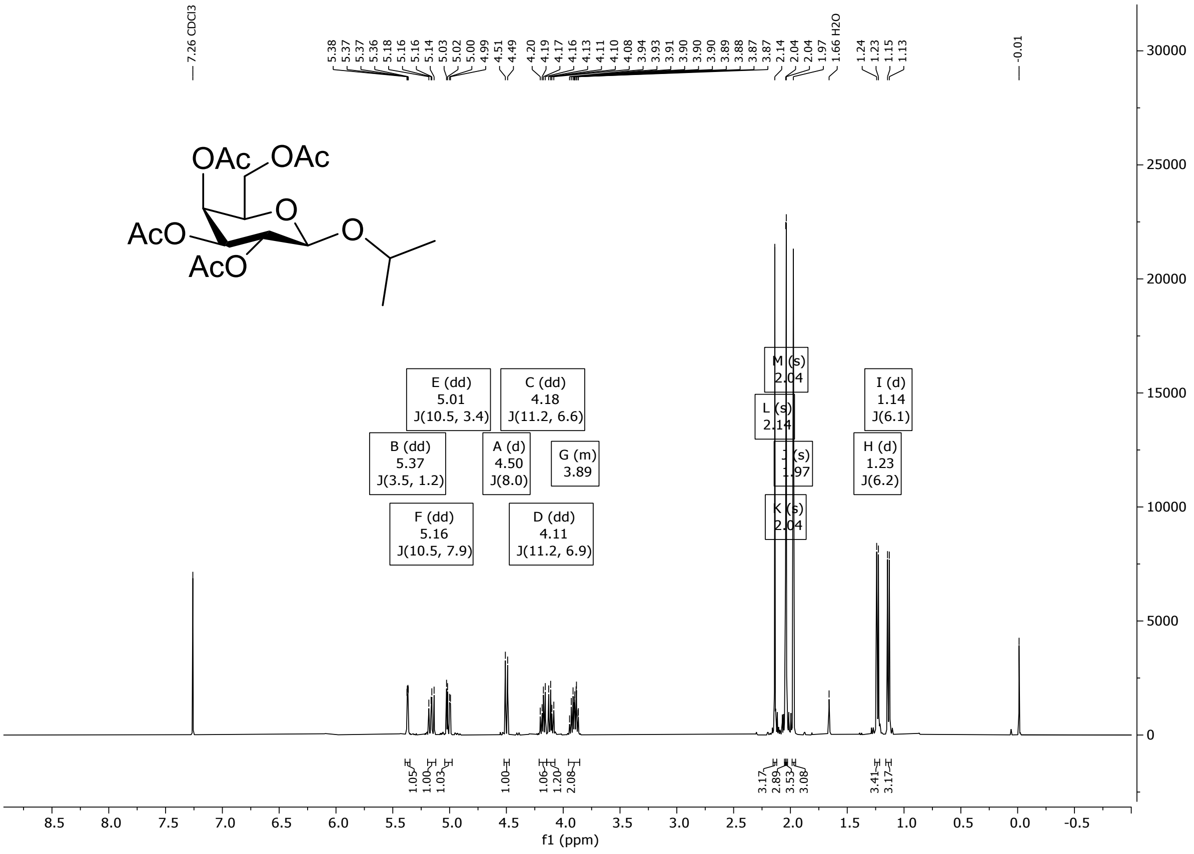

Figure S28. ^{1}**H NMR spectrum of crude isopropyl 2,3,4,6-tetra-*O*-acetyl-β-D-galactopyranoside** (400 MHz, $CDCl_3$).

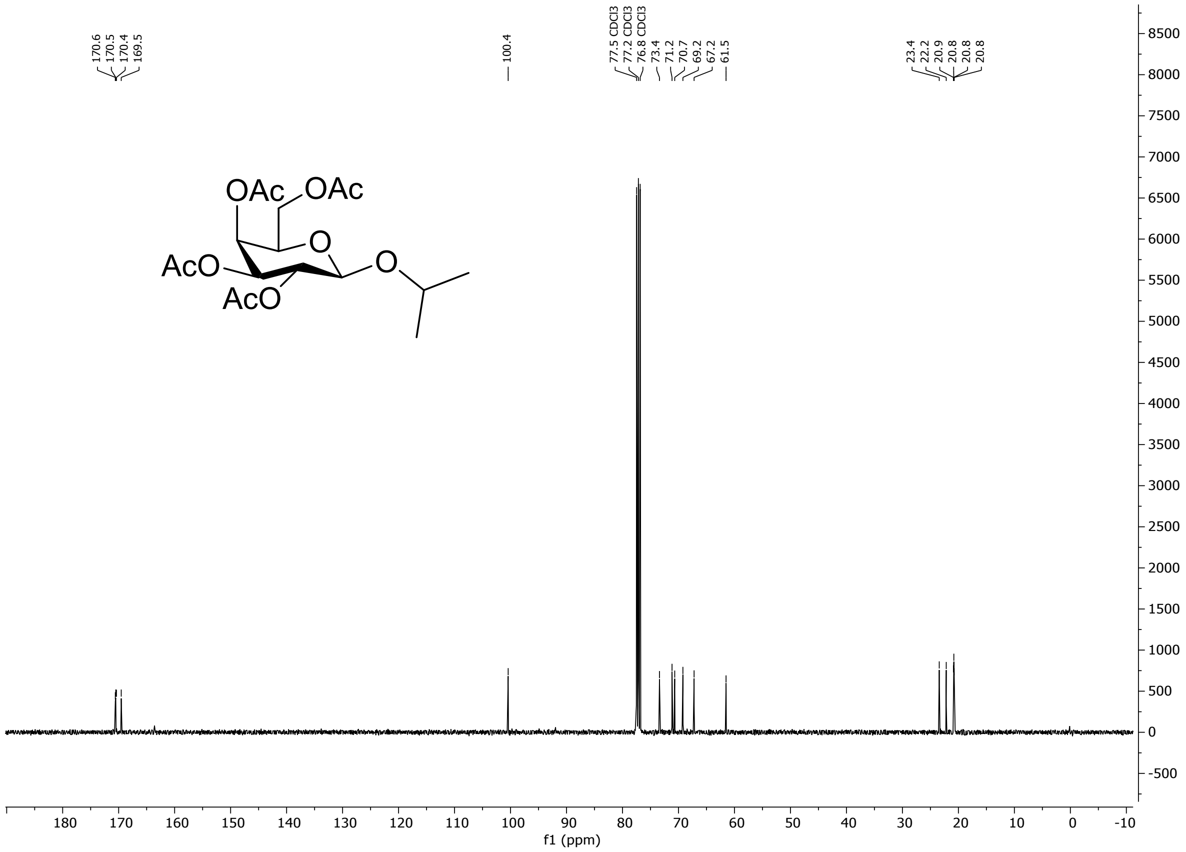

Figure S29. $^{13}C\{^{1}H\}$ **NMR spectrum of crude isopropyl 2,3,4,6-tetra-*O*-acetyl-β-D-galactopyranoside** (101 MHz, $CDCl_3$).

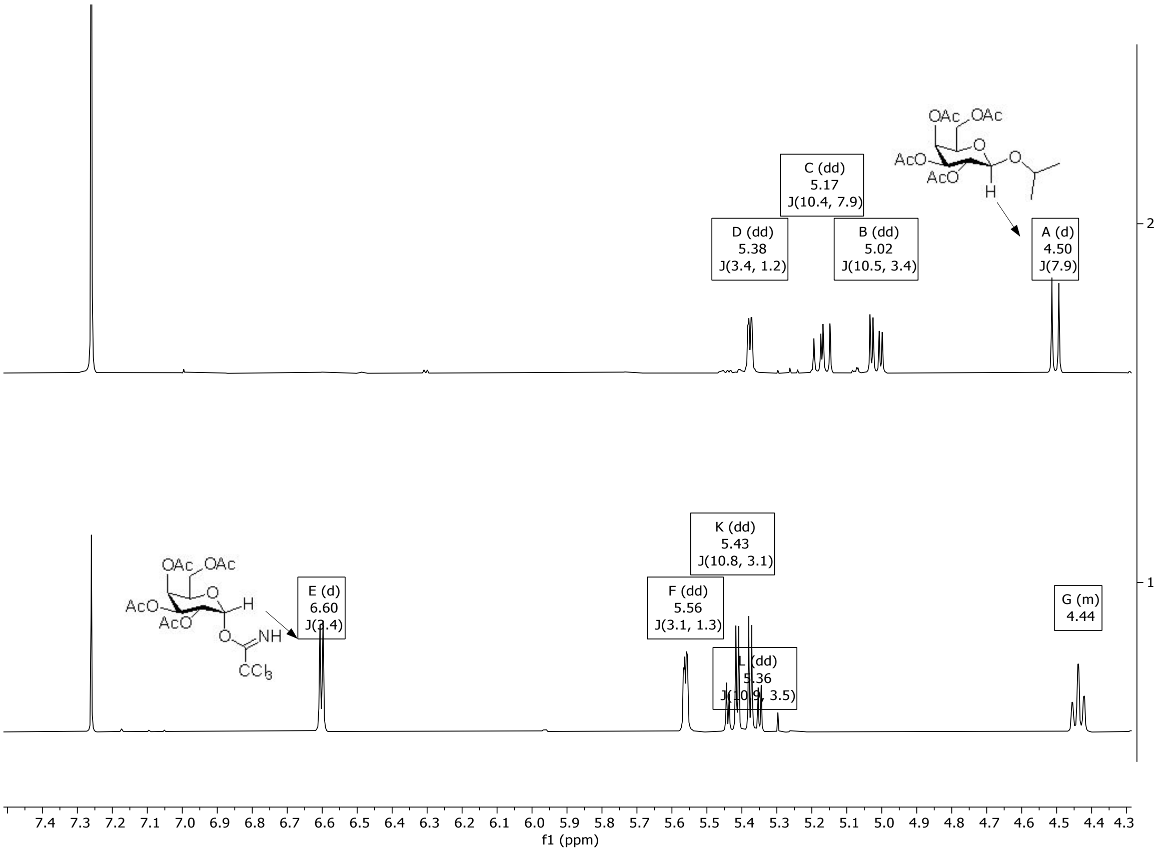

Figure S30. Glycosylation product formation in the crude reaction mixture monitored by ^{1}H NMR analysis (upper spectrum) compared to 2,3,4,6-tetra-*O*-acetyl-α-D-galactopyranosyl trichloroacetimidate (lower spectrum).

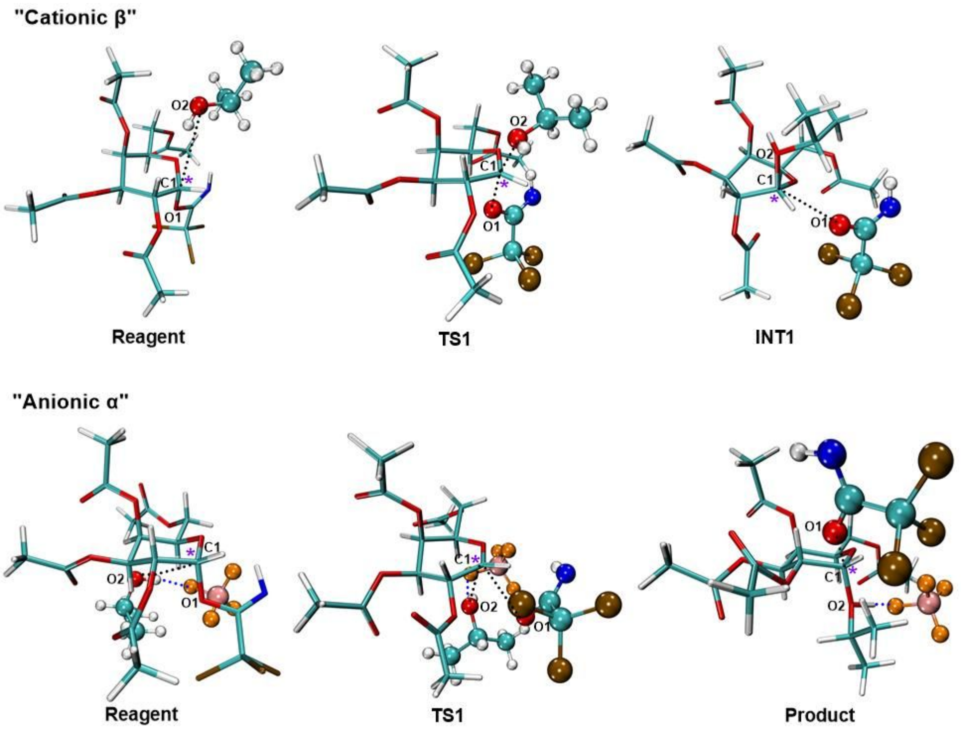

Figure S31. „Cationic β“ and „Anionic α“ pathways modelled to evaluate the performance of different DFT methods for the system under study.

Table S13. Gibbs free energies in kcal/mol calculated for „cationic β“ and „anionic α“ pathways using different methods.

[a] Reagent geometry optimization did not converge with this method. Used opt=cartesian keyword and additional optimization to locate the geometry.

[b] Since TS was not successfully located with the method, the geometry obtained with the B3LYP/6-31+G(2d,2p) method was reoptimized instead to obtain TS energy.

Basis set	Functional	TS-β	INT-β	TS-α	INT-α
**6-31+G (2d,2p)**	**B3LYP-D3(BJ)**	51.9	29.1	33.0[a]	14.0
	**PBE0-D3(BJ)**	51.2	31.7	36.2	13.5
	**ωB97XD**	54.0	31.8	31.3[a]	15.7
	**M06-2X**	50.3	33.3	38.9	15.9
	**MPW1PW91-PFD**	49.3	30.4	42.4[a]	14.5
**Def2-SVP**	**B3LYP-D3(BJ)**	55.1	35.1	30.6[b]	9.2
	**PBE0-D3(BJ)**	52.2	34.5	36.1[b]	12.5
	**ωB97XD**	54.8	35.3	30.4	12.4
	**M06-2X**	53.4	35.7	35.4	13.7
	**MPW1PW91-PFD**	50.5	33.2	41.6	17.0

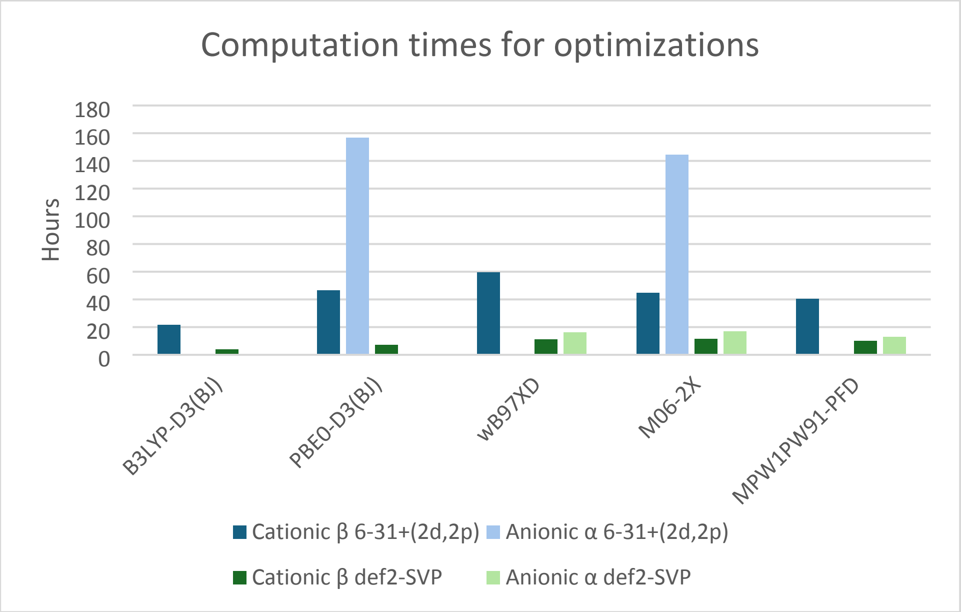

a)

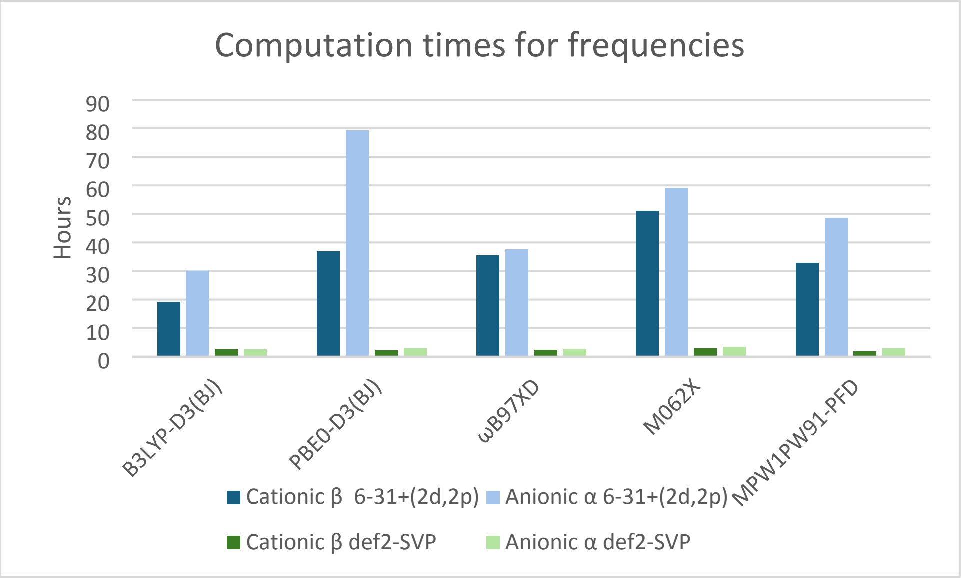

b)

Figure S32. a) Optimization and b) frequency calculation times for two different pathways with different methods.

Supplementary references

(1) Ghorai, J.; Almounajed, L.; Noori, S.; Nguyen, H. M. Cooperative Catalysis in Stereoselective O- and N-Glycosylations with Glycosyl Trichloroacetimidates Mediated by Singly Protonated Phenanthrolinium Salt and Trichloroacetamide. *J. Am. Chem. Soc.* 2024, *146* (50), 34413–34426. https://doi.org/10.1021/jacs.4c10633.

(2) Addanki, R. B.; Moktan, S.; Halder, S.; Sharma, M.; Sarmah, B. K.; Bhattacharyya, K.; Kancharla, P. K. Exploiting the Strained Ion-Pair Interactions of Sterically Hindered Pyridinium Salts Toward SN2 Glycosylation of Glycosyl Trichloroacetimidates. *J. Org. Chem.* 2024, *89* (6), 3713–3725. https://doi.org/10.1021/acs.joc.3c02207.
